# Genomic characterization of a multidrug-resistant *Staphylococcus xylosus* from Ecuadorian open market avocados: food safety and public health implications

**DOI:** 10.3389/fmicb.2025.1629139

**Published:** 2025-07-31

**Authors:** Gabriela N. Tenea, Evelyn Angamarca

**Affiliations:** Biofood and Nutraceutics Research and Development Group, Faculty of Engineering in Agricultural and Environmental Sciences, Universidad Técnica del Norte, Ibarra, Ecuador

**Keywords:** *Staphylococcus xylosus*, genomic analysis, virulence factors, antibiotic susceptibility, food safety, avocado

## Abstract

**Introduction:**

Foodborne bacterial infections remain a critical global health challenge, exacerbated by the increasing prevalence of antimicrobial resistance (AMR). Misuse of antimicrobials in agriculture and inadequate food handling practices facilitate the spread of resistant bacteria across the human–animal–environment interface, a central concern of the One Health approach. Comprehensive understanding of microbial threats in food systems is vital for effective risk assessment and control. In this study, we report the first complete genome of a multidrug-resistant *Staphylococcus xylosus* strain, FFCShyA4, isolated from commercially sold avocados.

**Methods:**

Whole-genome sequencing and comparative genomics were employed for taxonomic classification and phylogenetic analysis. In silico tools identified antibiotic resistance genes (ARGs), virulence factors, mobile genetic elements (MGEs), CRISPR loci, genomic islands, and biosynthetic gene clusters (BGCs). *In vitro* assays assessed hemolysis, gelatinase activity, antibiotic susceptibility, and PCR-based gene detection.

**Results:**

The FFCShyA4 genome spans 3.09 Mb with a 32.63% GC content and includes a 32 kb plasmid. It shares 99.97% average nucleotide identity with *S. xylosus* NBRC 109770 yet displays extensive structural rearrangements indicative of niche-specific adaptation. The genome encodes 2,720 protein-coding genes, including ARGs for β-lactams, macrolides, fluoroquinolones, tetracyclines, and lincosamides. The presence of 133 MGEs, CRISPR systems, an intact prophage, and 138 genomic islands reflects a strong potential for horizontal gene transfer. Virulence profiling identified 121 genes across 34 families, with a predicted human pathogenicity of 98.2%. BGCs linked to bacteriocins, siderophores, and staphylopine biosynthesis were also detected. *In vitro* assays confirmed multidrug resistance and pathogenicity.

**Conclusion:**

These results emphasize the critical need for integrated One Health surveillance of antimicrobial resistance within food production and commercial environments to facilitate early detection and reduce dissemination of resistance determinants across interconnected human, animal, and environmental reservoirs.

## 1 Introduction

*Staphylococcus xylosus* is a Gram-positive, coagulase-negative bacterium that primarily acts as a commensal on the skin and mucous membranes of mammals. It also adapts well to diverse environmental niches, such as soil, water, and surfaces linked to animal husbandry and food processing environments (Schiffer et al., 2023). Highly adaptable, *S. xylosus* can persist in challenging conditions, such as biofilms, high-salt environments, and low oxygen levels (Condas et al., 2017). It plays a significant role in food fermentation, contributing to the production of fermented meat products and specific cheeses by enhancing flavor development, stabilizing color, and ensuring safety through enzymatic activity and the production of antimicrobial metabolites (Leroy et al., 2017). Although primarily considered non-pathogenic, it has been associated with opportunistic infections like bovine mastitis and is recognized as a reservoir for antibiotic resistance genes and virulence factors, raising concerns about HGT to more pathogenic species such as *S. aureus* (Condas et al., 2017). Furthermore, growing evidence underscores the potential of coagulase-negative staphylococci (CNS) to act as reservoirs for virulence-associated factors (Marincola et al., 2021). Besides, *S. xylosus* is a rare colonizer of human skin, but it is more frequently found on the skin of individuals who have regular contact with animals (Battaglia and Garrett-Sinha, 2023). When human skin is transplanted onto nude mice, *S. xylosus* can be isolated from the grafts, though it colonizes a smaller proportion of grafts compared to the host mouse skin. This suggests that certain characteristics of murine skin support the colonization of *S. xylosus*, whereas human skin traits may prevent it (Kearney et al., 1982). While the most common staphylococcal species on human skin are part of the Epidermidis–Aureus group, *S. xylosus*, which is less prevalent, falls within the Saprophyticus sub-group (Lamers et al., 2012). Early genomic and *in situ* analyses of *S. xylosus* in a meat model reveal its adaptation to meat substrates, supported by genes enabling the utilization of diverse carbon, energy, and nitrogen sources (Dordet-Frisoni et al., 2007; Leroy et al., 2017). Its genome encodes pathways for osmotic, oxidative/nitrosative, and acidic stress responses, nitrate reductase activity for cured meat coloration, and enzymes for pyruvate and amino acid catabolism, contributing to odorous compounds (Labrie et al., 2014). In another study, a multidrug-resistant strain of *S. xylosus* NM36 was isolated from a cow with chronic mastitis (Al-Tameemi et al., 2023). This strain exhibited resistance to methicillin, ampicillin, cefoxitin, oxacillin, and tetracycline but remained susceptible to vancomycin, alongside a strong biofilm-forming capacity (Al-Tameemi et al., 2023).

While existing literature emphasizes the ecological specialization of *S. xylosus* in animal-related environments, a recent study revealed the presence of *S. xylosus* in fermented soybean foods in Korea (Kong et al., 2022). Most strains were susceptible to multiple antibiotics, but 23 showed potential acquired resistance to erythromycin, lincomycin, and tetracycline. Recently, we detected several *Staphylococcus* species on the exocarp of avocado fruits sold in open markets in Ecuador (Angamarca et al., 2023). Among these, *S. xylosus* FFCShyA4 exhibited antibiotic resistance. The presence of *S. xylosus* on the fruit exocarp can be explained by its ability to thrive in diverse environments, including plant surfaces, soil, and food-processing settings. This bacterium is known for its resilience, forming biofilms and utilizing various carbohydrates, which may facilitate its survival on the fruit surface. Thus, the presence of antibiotic-resistant bacteria in ready to eat fruits raises public health concerns (Rahman et al., 2021). Open markets are environments where both human and animal interactions are common, often facilitating the spread of bacteria between different sources (Tenea et al., 2023). However, it highlights the potential for these bacteria to spread from surfaces contaminated by animal products or human handling (Angamarca et al., 2023). These bacteria can then be transferred to consumers through direct contact with the food or improper handling during preparation. This scenario underscores the importance of proper hygiene and food safety measures to limit the spread of antibiotic-resistant bacteria and prevent potential health risks to consumers. To date, there are no reports on the genome of *S. xylosus* isolated from sources other than animals. In this study, we performed a comprehensive genomic analysis of the FFCShyA4 strain. The isolate was taxonomically classified, and its phylogenetic relationship with closely related strains was established. Functional genome mining was conducted to predict antibiotic resistance genes, virulence factors, CRISPR systems, MGE, and secondary metabolite biosynthesis. Additionally, *in vitro* assays were carried out to assess hemolytic activity, gelatinase production, and susceptibility to various antibiotics. These findings enhance our understanding of the genomic diversity, evolutionary dynamics, and public health risks associated with *S. xylosus*. By highlighting its potential role in foodborne contamination and cross-contamination, this study supports the development of targeted interventions to strengthen food safety systems and protect public health.

## 2 Materials and methods

### 2.1 Bacterial strain cultivation and DNA extraction

*S. xylosus* FFCShyA4 was isolated from the exocarp of avocado (*Persea nubigena* var. *guatemalensis*) as described by Angamarca et al. (2023). The strain was cultured in Brain Heart Infusion (BHI) broth (Merck Millipore, MA, USA). Genomic DNA and total RNA were extracted using the Illumina DNA Prep Kit (Illumina Inc., San Diego, CA, USA), following quality control procedures. DNA concentration was measured using a NanoDrop™ 2000 spectrophotometer (Thermo Fisher Scientific, USA), and libraries were prepared according to the manufacturer’s protocol.

### 2.2 *De novo* assembly and workflow sequencing

Genome sequencing was conducted using the Illumina HiSeq X Ten platform (Macrogen Inc., Seoul, Korea). Libraries were prepared by fragmenting DNA or cDNA samples and ligating 5′ and 3′ adapters, as per the manufacturer’s protocol. Adapter-ligated fragments were PCR-amplified, gel-purified, and loaded onto a flow cell for cluster generation via bridge amplification or ExAmp cluster amplification on patterned flow cells. Sequencing was performed using Illumina SBS technology, a reversible terminator-based method that minimizes incorporation bias and reduces sequence errors, even in repetitive or homopolymer regions, by ensuring natural competition among all four dNTPs. Raw sequencing data were analyzed, and FastQC (v0.11.5) was used to assess quality, base count, GC content, and other metrics. *De novo* assembly was carried out with SPAdes v3.15.5 using multiple k-mers, and the best assembly was chosen based on parameters like contig number, total contig bases, and N50 (Bankevich et al., 2012). Assembly completeness was evaluated using BUSCO v5.1.3 against the bacterial or eukaryotic database, based on near-universal single-copy orthologs. BLAST analysis was used to identify the species corresponding to each scaffold.

### 2.3 Typing and species relatedness

ANI analysis was performed by aligning the reference sequence (Taxon ID: GCF_007992815.1_*S. xylosus_*NBRC_109,770) with contig 1 using BLASTN (custom assay project, Macrogen Inc., Seoul, Korea). A circular genome map was generated via the PROKSEE server^1^ (Grant et al., 2023). Genome sequence data underwent further analysis through the Type (Strain) Genome Server (TYGS) for a comprehensive genome-based taxonomic study (Meier-Kolthoff and Göker, 2019). The closest related strain genomes were identified using MASH distance comparisons, with precise distances calculated by the Genome BLAST Distance Phylogeny (GBDP) method under the “coverage” algorithm (Meier-Kolthoff et al., 2013). Phylogroups were classified using *in silico* Clermont Phylotyper EzClermont (Waters et al., 2020). Synteny analysis was conducted via multiple genome alignment using Mauve with default parameters (minimum Localized Collinear Blocks (LCBs) of 1,000, island size of 50, backbone size of 50, and maximum gap of 50). The draft genome of FFCShyA4 was aligned relative to the *S. xylosus* CCM2738 and *S. xylosus* NBRC 109770, reference genomes—originally isolated from human skin (Darling et al., 2004).

### 2.4 General genome features, gene prediction, and functional annotation

The identification of coding sequences (CDS), rRNA, tRNA/tmRNA, signal leader peptides, and noncoding RNA was conducted following the methodology outlined by Molina et al. (2024). Gene annotation was performed using Prokka v1.14.6 (Seemann, 2014), while plasmid annotation was carried out with pLannotate (McGuffie and Barrick, 2021). Functional annotation was achieved using InterProScan v5.0 (Jones et al., 2014), which evaluates sequence similarity at the family level and matches sequences against member databases within InterPro, including Pfam, the Conserved Domain Database (CDD), and TIGRFAM, a curated database focusing on prokaryotic protein families (Selengut et al., 2007). Furthermore, the EggNOG database (Evolutionary Genealogy of Genes: Non-supervised Orthologous Groups) was utilized for additional annotations (Huerta-Cepas et al., 2019), with psi-BLAST applied to align predicted protein sequences to the EggNOG database.

### 2.5 *In silico* genome analysis

#### 2.5.1 Prediction of CRISPR sequences, prophage, antibiotic resistant genes (ARGs), virulence factors (VFs), genomic island (GIs), genetic mobile elements (MGEs), and pathogenicity

To identify CRISPR, Cas sequences, and prophage regions in bacterial genomes, CRISPRCasFinder (Crispr-Cas++1.1.2) and the PHAge Search Tool Enhanced Release (PHASTER) were employed (Arndt et al., 2016). The CARD (Comprehensive Antibiotic Resistance Database) tool and the RGI (Resistance Gene Identifier) were used to detect ARGs, applying Perfect hit, Rigorous hit, and Loose hit criteria (Jia et al., 2017; Arndt et al., 2016). To identify acquired ARGs, the ResFinder 4.1 server was utilized with a 90% ID threshold and a minimum length of 60% (Zankari et al., 2012), alongside the detection of chromosomal mutations (Bortolaia et al., 2020). Putative virulence factors were predicted using the VFDB database (Liu et al., 2019). Genomic islands were predicted using the IslandViewer 4 server (Bertelli et al., 2017) and bacterial pathogenicity was assessed via the PathogenFinder web server (Cosentino et al., 2013). MGEs were predicted using mobileOG-db tool (Brown et al., 2022) incorporated in the PROKSEE server (see text footnote 1).

#### 2.5.2 Prediction of bacteriocin encoding genes clusters and secondary metabolites

For the detection of biosynthetic gene clusters (BGCs) of antimicrobial compounds, the web tool BAGEL 4 (van Heel et al., 2018) was used. The secondary metabolites prediction was assessed using antiSMASH version 4 webtool (Blin et al., 2023).

### 2.6 *In vitro* analysis

#### 2.6.1 Hemolysis and gelatinase production

Hemolysin production in the FFCShyA4 isolate was assessed by inoculating the strain onto 5% human blood agar plates, followed by incubation at 37°C for 24 h, as described by Tabasi et al. (2015). Hemolytic activity was evaluated based on the presence of partial or complete zones of erythrocyte lysis surrounding the bacterial colonies. Gelatinase production was determined using gelatin nutrient agar plates, following the protocol established by Mittal et al. (2014). An overnight culture of the isolate was inoculated onto the plates, and after visible growth, the medium was treated with a mercuric chloride solution. A clearing zone surrounding the bacterial colonies, resulting from gelatin hydrolysis, indicated a positive gelatinase activity. *S. aureus* ATCC43300 and *S. aureus* ATCC1026 were included as controls in both assays.

#### 2.6.2 Antibiotic susceptibility evaluation

Antibiotic susceptibility was determined using the Muller-Hilton (MH) agar disk diffusion procedure, and according to the Clinical and Laboratory Standards Institute (CLSI) guidelines (Clinical and Laboratory Standards Institute, 2021). Briefly, 100 μL of inoculum (10^7^–10^8^ CFU/mL) was streaked onto MH plates. The commercial antibiotic disks (Merck, USA) chosen are listed in Supplementary Table S1A. The disks were plated on MH agar plates, and incubated at 37°C for 48 h. The diameter of each clear zone was measured in millimeters by scanning the plates with a microplate reader (SCAN500, Interscience, Fr). As controls, *S. aureus* ATCC43300 and *S. aureus* ATCC1026 were included in the experiments. The microbiological breakpoints reported by Clinical and Laboratory Standards Institute (2021) and the European Committee on Antimicrobial Susceptibility Testing (2021) standards were used to categorize *Staphylococcus* as susceptible (S), intermediate (I), or resistant (R) (Supplementary Table S1B). The MAR index was also determined as a ration between the total number of antibiotics to which the isolate is resistant and the total number of antibiotics tested.

#### 2.6.3 Virulence and antibiotic genes detection by PCR

Primers targeting virulence genes thermonuclease (*nuc*), intracellular adhesin (*ica*A), putative adhesin (*sdr*E), hemolysin (*hlg*), meticillinA (*mec*A), and meticillinC (*mec*C) (Stegger et al., 2012) were prepared at a concentration of 0.3 μM (Supplementary Table S2). Genomic DNA was extracted using the Wizard® Genomic DNA Purification Kit (#1120, Promega, USA). DNA concentration and purity were measured using a NanoDrop™ spectrophotometer (Thermo Fisher Scientific, USA) at absorbance wavelengths of 230, 260, and 280 nm. PCR amplifications were carried out in 25 μL reaction volumes, each containing 2X GoTaq® Green Master Mix (#7132, Promega, USA). Reactions were performed using a Genemax Thermal Cycler (IQM, Oslo, Norway). The amplification conditions are shown in Supplementary Table S2. PCR products were separated on 1% agarose gels in 1X Tris-Borate-EDTA (TBE, pH 8.0) buffer (Sigma-Aldrich, USA). The gels were stained with TBE buffer containing 0.5 μg/mL ethidium bromide. Results were recorded as positive or negative based on the presence or absence of the expected amplicons.

## 3 Results and discussion

### 3.1 General genome characteristics, taxonomy, and phylo-genetic relationship

**Figure 1:**
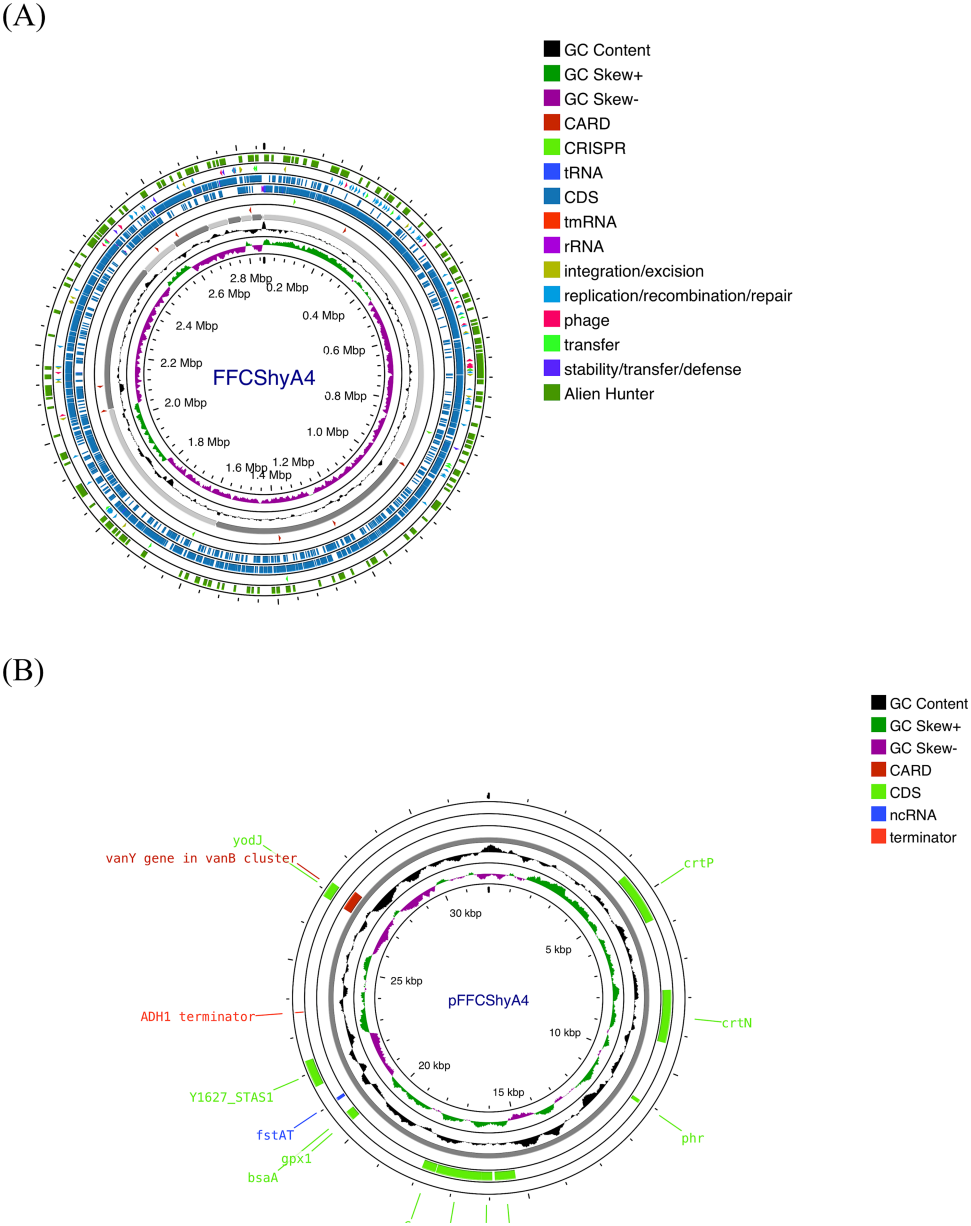
**(A)** Genome map of *S. xylosus* FFCShyA4 predicted with the PROKSEE viewer (Accessed on January 20, 2025). **(B)** Plasmid map as predicted by pLannotate. The contents are arranged in feature rings. Starting with the outermost ring: ring 1, mobile genetic elements (MGE) annotation with Alien Hunter predicting HGT (Horizontal Genetic Transfer) events (in red color); ring 2, MGE annotation with Mobile OG DB marking the *hsdR* gene involved in stability/transfer/defense; rings 3–4 show the CDS (protein-coding genes) with Prokka annotation (blue color), tRNA, rRNA, and tmRNA are marked; ring 5 displays the CRISPR-Cas annotation; ring 6, CARD annotation, ring 7 GC content plot (black); ring 8 displays G/C skew information in the (+) strand (green color) and (−) strand (purple color).

**Figure 2:**
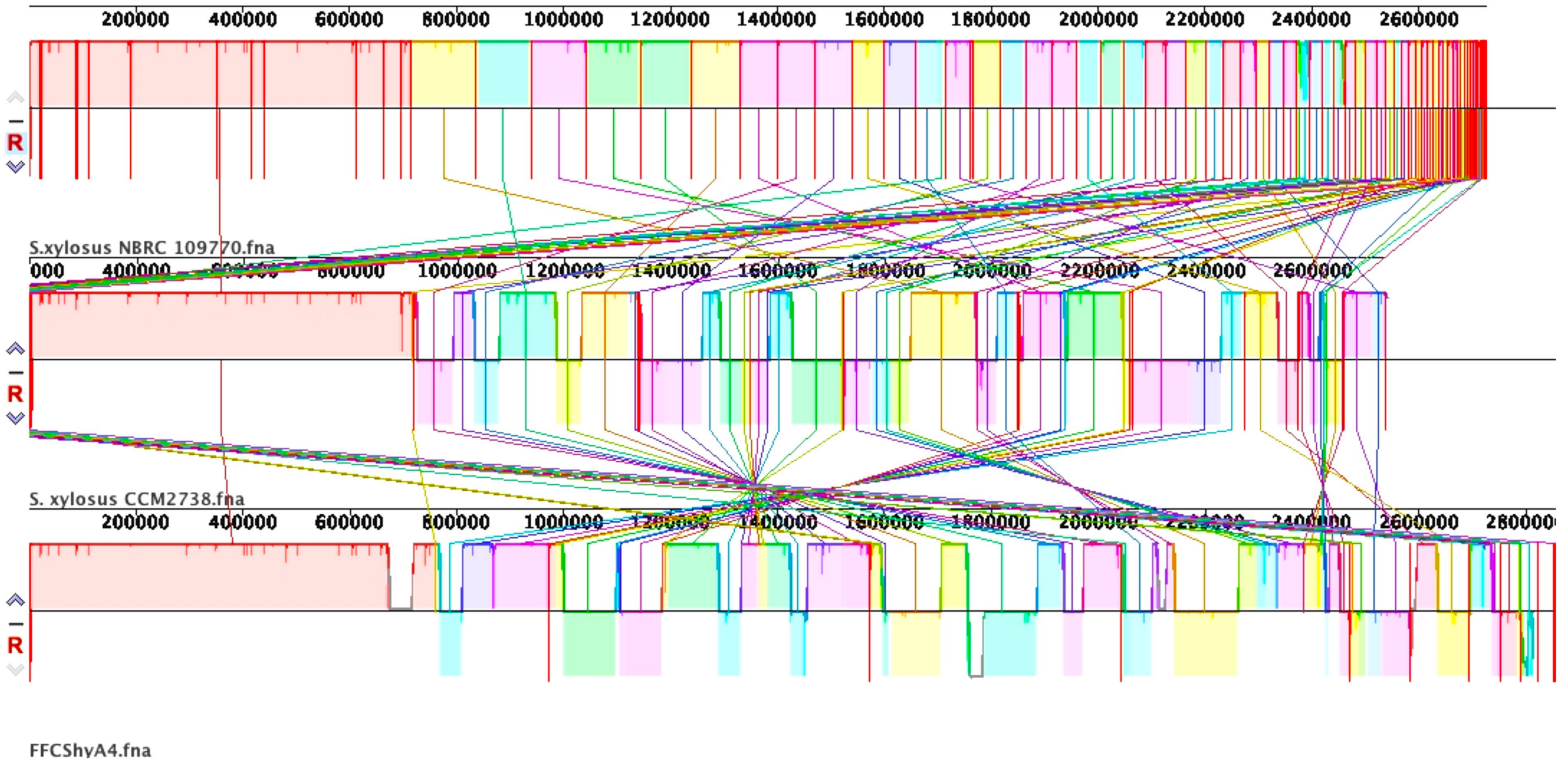
Whole-genome alignment of FFCShyA4 and references strains *S. xylosus* CCM2738 and *S. xylosus* NBRC109770 using Mauve Contig Mover. Colored blocks represent local collinear blocks (LCBs) with highly conserved regions between the genomes. Crossing lines indicate genomic rearrangements, while the absence of LCBs in specific regions suggests strain-specific sequences.

For the FFCShyA4 strain, a total of 2,972,650,628 bases were generated, producing 19,686,428 pair-end reads. The total number of contigs was 2,856,035 bp. The GC content was calculated at 32.63%, with a Q30 value of 92.75%. The estimated genome size was 3,093,530 bp. In addition, the strain harbored one plasmid of 32,035 bp. The general genome features of FFCShyA4 are shown in Supplementary Table S3. The circular genome and plasmid maps are shown in Figures 1A,B. Following assembly of the complete or draft genome, BLAST analysis was conducted to identify species-level similarities for each scaffold. Genus-level classification revealed that 90.91% of the scaffolds matched *Staphylococcus*, while 9.09% aligned with *Mammaliicoccus*. ANI analysis revealed a 99.97% nucleotide identity and 94.19% alignment coverage with *S. xylosus* NBRC109770 (human skin) (Lamers et al., 2012). The five most closely related genomes identified based on ANI results are presented in Supplementary Table S4. Hierarchical clustering of ANI data was visualized as two-dimensional dendrograms, using simple linkage for both ANI percentage identity and ANI alignment coverage (Supplementary Figures S1A,B). BLASTN analysis revealed that plasmid pFFCShyA4 shares 99.26% sequence identity with plasmid pDMSX03-1 from *S. xylosus* strain DMSX03. Phylogenetic analysis identified *S. xylosus* strain CCM2738 and *S. pseudoxylosus* strain S044009 as the closest related strains as shown by TYGS analysis (Supplementary Figure S2). The Mauve alignment of FFCShyA4, and the two references *S. xylosus* NBRC109770 and *S. xylosus* CCM2738 reveals a mix of conserved and variable regions, highlighting both evolutionary stability and divergence (Figure 2). The conserved regions, mainly found at the genomes terminal ends and encompassing essential housekeeping genes, suggest a high degree of preservation among the three strains (González et al., 2010). However, extensive genomic rearrangements, including inversions and translocations, are observed between 1,000,000–1,800,000 bp, suggesting evolutionary divergence (Shukla et al., 2009). Notable insertion/deletion (indel) events occur within 600,000–1,000,000 bp and 2,200,000–2,600,000 bp, with FFCShyA4 displaying unique genomic islands absent in the reference strains, potentially linked to adaptation in food-related environments (Vermassen et al., 2016). Additionally, the high density of intersecting lines observed in the 1,200,000–1,600,000 bp region indicates potential hotspots for horizontal gene transfer (HGT), likely involving MGEs (Ghaly et al., 2024). These regions frequently harbor genes linked to antibiotic resistance, responses to metal and oxidative stress (e.g., copper and arsenic resistance genes, superoxide dismutase), as well as carbohydrate metabolism and fermentation. The presence of HGT-associated markers, including transposases and prophage-related genes, underscores the strain’s metabolic versatility and genomic plasticity (Coll et al., 2025). However, the alignment highlights conserved core regions while also identifying unique genomic segments in FFCShyA4 that may contribute to its distinct functional capabilities, including potential virulence and resistance traits (Zhou et al., 2020). These results support the hypothesis that FFCShyA4 shares a common lineage with NBRC 109770 and CCM2738 but has undergone genetic modifications that could enhance its survival in food-associated environments or human-related settings, warranting further investigation into its role in public health and food safety (Vermassen et al., 2014). These findings suggest that the FFCShyA4 strain may possess unique traits enhancing its environmental adaptability and resilience, particularly in response to stressful or competitive conditions such fruit environment.

### 3.2 Gene prediction and functional annotation

**Figure 3:**
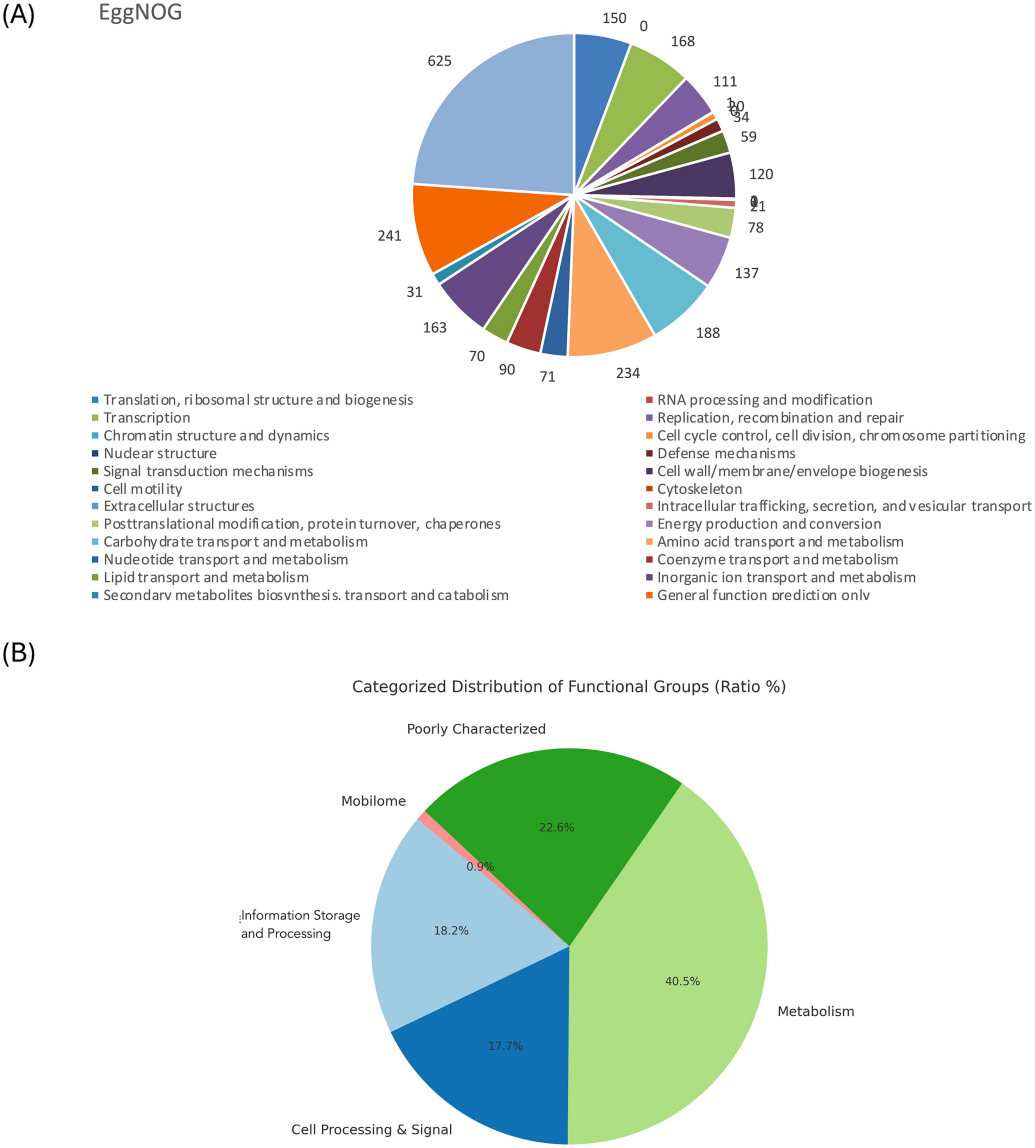
**(A)**. EggNOG category distribution of functional annotation result. **(B)**. Pie chart showing the COG distribution of the overall categories.

The genome comprises 2,720 genes, including 2,665 coding sequences (CDS), 47 tRNAs, 7 rRNAs, and 1 tmRNA (Supplementary Table S3). Gene locations were predicted using Prokka, while BLAST analysis was performed to determine the functions and identify the assembled sequences by comparing them against nucleotide and protein sequence databases. The predicted genes were subsequently aligned with multiple databases to obtain functional annotations using specific aligners, as summarized in Table 1. Among the total proteins, 2,576 matched EggNOG DB proteins (2,538 Single EggNOG and 38 Multi EggNOG proteins) and 89 proteins with no hit (Figure 3A). Similarly, 2,476 proteins matched COG DB (2,210 Single COG category and 266 Multi COG category) while 189 showed no hit (Figure 3B). The number of genes associated with KEGG (2,580 genes) functional annotation categories is shown in Supplementary Figure S3. The plasmid annotation (Figure 1B) includes several key features: *ars*B (100% identity) from *S. xylosus*, involved in arsenical resistance and likely forming the channel of an arsenite pump; *ars*R (99% identity) from *S. aureus*, a transcriptional repressor for the *ars* operon, which regulates its own expression and is derepressed by oxyanions of arsenic, antimony, and bismuth in the +III oxidation state, as well as arsenate (As(V)); *ars*C (95.4% identity) from *S. aureus* strain N315, encoding an enzyme that reduces arsenate [As(V)] to arsenite [As(III)]; *bin*3 (83.2% identity) from *S. aureus*, a potential DNA invertase; *crt*N (66.5% identity) and *crt*P (65% identity) from *S. aureus*, involved in staphyloxanthin biosynthesis, a carotenoid contributing to virulence by protecting against oxidative stress; and ADH1 terminator (100% identity), a transcription terminator for the *S. cerevisiae* alcohol dehydrogenase 1 (ADH1) gene. These features highlight roles in resistance, enzymatic activity, and oxidative stress protection. ADH1 is not naturally found in *Staphylococcus* species (Makhlin et al., 2007). In the context of plasmid annotation, the ADH1 terminator is used as a transcription terminator, and possible serves as a genetic element for controlling gene expression.

**Table 1:** Gene annotation summary.

Num of genes (#)	CARD	MetaCyc	CAZy	PHI	VFDB	SwissProt	KEGG	COG
2,671	61 (2.28%)	539 (20.18%)	92 (3.44%)	289 (10.82%)	122 (4.57%)	1,481 (55.45%)	2,580 (96.59%)	2,104 (78.77%)

CARD, Comprehensive Antibiotic Resistance Database; MetaCyc, database that contains pathways involved in both primary and secondary metabolism (Caspi et al., 2018); PHI, The Pathogen-Host Interaction database is a biological database that contains curated information on genes experimentally proven to affect the outcome of pathogen-host interactions (http://www.phi-base.org/searchFacet.htm?queryTerm); CAZy, Carbohydrate-active enzyme (http://www.cazy.org/); VFDB, virulence factor database, http://www.mgc.ac.cn/VFs/main.htm; SwissProt: https://www.uniprot.org/uniprotkb/statistics; KEGG, Kyoto Encyclopedia of Genes and Genomes; COG, Clusters of Orthologous Groups of proteins.

### 3.3 Prediction of CRISPR sequences, prophage, ARGs, VFs, GIs, MGEs, and pathogenicity

Three CRISPR sequences were identified in the FFCShyA4 genome, located at positions 79,658 to 79,743, 868,840 to 868,928, and 47,184 to 47,284, respectively. Each sequence includes a short spacer sequence flanked by degenerate repeats (consensus DRs), with repeat conservation of 96.68, 96.65, and 100%, and spacer conservation of 100%. No Cas genes were found. One intact prophage was found in the genome (Supplementary Table S5). The detection of Phage *Staphy_47* in the FFCShyA4 genome is significant as it highlights the potential influence of prophages on the genetic and functional diversity of the host bacterium. This phage can contribute to HGT, providing the host with adaptive advantages, including virulence factors, resistance genes, or enhanced survival mechanisms under stress conditions (Deghorain and Van Melderen, 2012). Additionally, a total of 138 GIs of 126,044 bp were predicted with IslandViewer using as a reference genome of non-coagulase *S. xylosus* strain DMSX03 isolated from fermented soybean (Heo et al., 2021). Many hypothetical proteins and enzymes, such as proteases, and virulence factors (*maz*E, *Pem*K/*Maz*F), were identified in the GIs. These genomic regions are crucial for the rapid evolution, diversification, and adaptation of *Staphylococcus* due to their frequent rearrangements, excisions, transfers, and the acquisition of additional DNA (Supplementary Table S6). Among the 121 genes identified from the virulence database, the thermonuclease gene (*nuc*), the intercellular adhesion protein C involved in polysaccharide intercellular adhesin (PIA) synthesis (*ica*C), and the ATP-dependent Clp protease proteolytic subunit (*clp*P) were detected, although they exhibited imperfect matches (identity < 85%, with the input sequence length shorter than the full virulence gene length, and 60% alignment coverage). In addition, the elongation factor Tu (*tuf*A) and phosphopyruvate hydratase (*eno*) were annotated showing 74 and 79.8% sequence identity, respectively. The detected virulence factors are summarized in Supplementary Table S7. Interesting, the presence of *nuc* but not *icaC, sdrE,* and *hlg* genes was confirmed by PCR (Supplementary Figure S4), as these genes typically are associated with *S. aureus*. The detection of the *nuc* gene in CNS strain may be attributed to HGT, genomic variability within CNS, or the presence of a divergent gene variant with partial homology, highlighting the potential for genetic exchange and adaptation in diverse environments (Canning et al., 2020). Besides, several virulence factors such as surface-displayed alpha-enolase (*eno*), adaptor protein MecA (*mec*A), type II toxin-antitoxin system PemK/MazF family toxin, a virulence associated protein E (*vir*E), were annotated with EggNOG. Moreover, within FFCShyA4 genome, a total of 133 MGEs were identified, including 17 related to insertion/excision, 74 associated with replication/recombination/repair, 21 linked to phages, 6 involved in stability/transfer/defense, and 15 related to transfer. Analysis of the 34 matched pathogenic families (accounting for 1.28% of the proteome) indicated that the FFCShyA4 isolate is a human pathogen with a likelihood of 0.982. This finding was further supported by the presence of an inhibition zone on blood agar, indicative of hemolytic activity (Supplementary Figure S5). Additionally, the gelatinase test for FFCShyA4 was positive, consistent with previous research showing that gelatinase is a common virulence factor among *Staphylococcus* spp. (Bertelloni et al., 2021). Using CARD protein IDs, 61 predicted genes were classified by drug class, resistance mechanism, and AMR gene family, with the distribution of genes shown in Supplementary Figure S6. Besides, 11 ARGs including vancomycin (*van*Y gene in *van*A cluster), lincosamide (*sal*D), and fosfomycin (*fos*Bx1) resistance were detected according to the RGI tool criteria. The presence of acquired ARGs indicates that this strain was likely exposed to selective pressures, such as environmental antimicrobial use, which may have promoted the acquisition of these resistance genes through horizontal gene transfer (HGT) or other mechanisms (Chen et al., 2022). Based on EggNOG annotation, we confirmed the presence of several key antibiotic resistance-related genes in the FFCShyA4 genome, including *fus*A*, pbp*4, and *ile*S. The *fus*A gene encodes elongation factor G (EF-G), which is essential for protein synthesis and serves as a target for antibiotics like fusidic acid, suggesting a potential mechanism for resistance to this antibiotic (Resch et al., 2008). The *pbp*4 gene encodes penicillin-binding protein 4 (PBP4), which plays a critical role in bacterial cell wall synthesis and is a key target of β-lactam antibiotics like penicillin (Satishkumar et al., 2021). Alterations in PBP4 can reduce the binding affinity of these antibiotics, contributing to resistance. However, its role in *S. xylosus* was not yet identified. Additionally, *ile*S, which encodes isoleucine-tRNA ligase, is involved in protein synthesis and may influence resistance indirectly by impacting bacterial growth and response to translation-targeting antibiotics (Grundy et al., 1997). These findings indicate that the FFCShyA4 strain exhibits a high level of adaptability to antibiotic pressure, its genomic features suggest it may serve as a reservoir of ARGs across environmental interface. These genomic features suggest that FFCShyA4 possesses ecological adaptability and may function as a reservoir of ARGs, with potential relevance to One Health pathways of AMR dissemination across human, animal, and environmental interfaces (Robinson et al., 2016).

### 3.4 BGCs organization predicted from genome study

**Figure 4:**
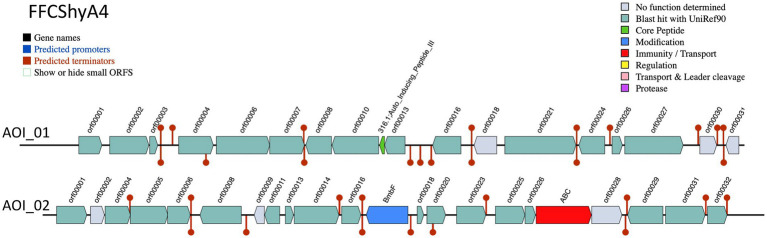
Bacteriocin cluster genes organization. Area of Interest (AOI) of FFCShyA4. Legend: red blocks: immunity and transport; green arrow: core peptide; blue block: peptide modifications; grey blocks: no function determined.

Two bacteriocin clusters (Area of interest, AOI) were annotated within the FFCShyA4 genome as follows: contig 5.2 (AOI_01) (started at 18217, end at 38349) of auto-inducing peptide III (AIP III) class, and contig 5.2 (AOI_02) (started at 85346, end at 105367) of the sactipeptides class (ribosomally synthesized peptides) (Figure 4). BLASTP analysis revealed that AIP III shares 100% amino acid sequence identity with the cyclic lactone autoinducer peptide AgrD found in several *Staphylococcus* species, including *S. xylosus*. The *agr* locus was originally characterized in *S. aureus* as a regulatory element that controls the production of exoproteins involved in virulence (Dufour et al., 2002). Besides, AIP III plays a critical role in quorum sensing within *Staphylococcus* species, particularly *S. aureus* (Tamai et al., 2023). By regulating gene expression in response to population density, AIP III influences virulence factors, biofilm formation, and antibiotic resistance. This peptide signals the bacteria to produce toxins and surface proteins that aid in immune evasion and infection persistence, while biofilms protect the bacteria from antimicrobials (Tamai et al., 2023). Disrupting AIP III-mediated quorum sensing presents a potential therapeutic strategy to reduce bacterial virulence and biofilm formation (Gonzales et al., 2024). In addition, the sactipeptides, a class of ribosomally synthesized and post-translationally modified peptides (RiPPs), exhibit diverse biological activities, including antibacterial, spermicidal, and hemolytic properties (Chen et al., 2021). Analysis of the FFCShyA4 genome using the antiSMASH web tool identified nine distinct biosynthetic regions (Table 2). These include a terpene biosynthetic cluster (region 1.1) with *heme* D1 as the closest known cluster (11% similarity), an NI-siderophore cluster (region 2.1) with 100% similarity to a known siderophore cluster, and regions encoding Type III polyketide synthase (T3PKS) and non-ribosomal peptide synthase (NRPS) on contigs 2 and 3. Based on gene similarity analysis with the MiBIG database, a 54% sequence similarity was observed with staphylopherrin A found in *S. aureus* subsp. *aureus* NCTC 8325 (Supplementary Table S8), which plays a vital role in iron acquisition (Battaglia and Garrett-Sinha, 2023). Staphylopherrin A aids the bacteria by binding to iron (Fe3+) in the environment and facilitating its transport into the bacterial cell (Battaglia and Garrett-Sinha, 2023). Early genomic analysis of the *S. xylosus* C2a strain reveals the presence of genes encoding staphylopherrin A (Vermassen et al., 2014). This iron acquisition mechanism enables *S. aureus* to survive and thrive within the host, enhancing its virulence and ability to cause infections. This suggests that *S. xylosus* may utilize similar mechanisms as *S. aureus* to acquire iron from the environment. Notably, one NRPS cluster predicted within FFCShyA4 genome shared 8% similarity with surfactin. Additionally, an opine-like metallophore cluster located on contig 3 displayed 100% similarity to the staphylopine biosynthesis cluster. Finally, a cyclic lactone autoinducer biosynthetic region (region 5) showed 4% similarity to the kijanimicin cluster. Staphylopine, a versatile metallophore produced by the prominent human pathogen *Staphylococcus aureus*, is essential for the uptake of transition metals and contributes significantly to bacterial virulence (Song et al., 2018). These findings suggest that *S. xylosus* may employ virulence-like strategies like *S. aureus*, underscoring the need to assess its role in antimicrobial resistance dissemination and cross-domain adaptation (Battaglia and Garrett-Sinha, 2023). Further research should explore the functional roles of the AIP III and sactipeptide bacteriocin clusters in FFCShyA4, particularly their involvement in quorum sensing, biofilm formation, and microbial competition within foodborne ecosystems.

**Table 2:** Identified secondary metabolite biosynthetic gene clusters with antiSMASH using strictness “strict.”

Contig. Region	Type	ClusterBlast*/KnownCluster Blast gene similarity (%)
1.1	Terpene	Heme D1/ 11
2.1	NI-siderophore	Staphyloferrin A/ 100
2.2	T3PKS	No determined
2.3	NRPS	Surfactin/ 8
3.1	Opine like metallophore/ terpene	Staphylopine/ 100
3.2	NRPS	No determined
3.3	NRPS	No determined
5.1	Cyclic-lactone-autoinducer	Kijanimicin/ 4
9.1	Terpene	No determined

^*^% similarity with several *Staphylococcus* strains from database.

### 3.5 Hemolysis, gelatinase, and antibiotic susceptibility tests *in vitro*

**Figure 5:**
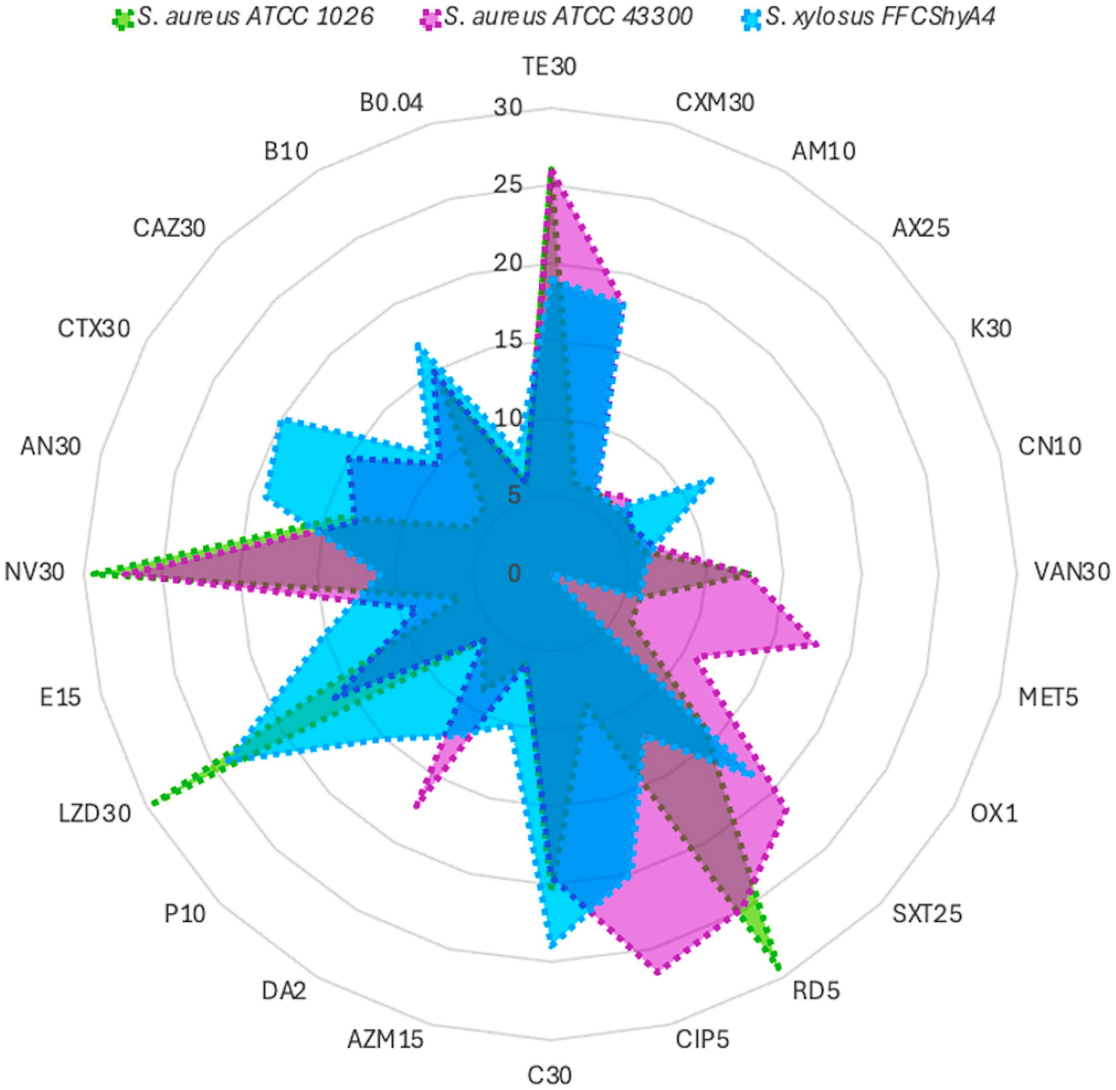
Antibiotic profile of *S. xylosus* FFCShyA4 compared with *S. aureus* ATCC1026 and *S. aureus* ATCC43300.

*S. xylosus* FFCShyA4 exhibited β-hemolytic activity and tested positive for gelatinase, like the *S. aureus* reference strains ATCC1026 and ATCC43300, indicating a potential adaptive trait rather than a direct virulence marker (Supplementary Figure S5). Genome annotation revealed the presence of a hemolysin III family protein in FFCShyA4; however, the failure to amplify hemolysin genes via PCR suggests potential sequence variations leading to primer mismatches or regulatory mechanisms influencing gene expression. Alternatively, hemolytic activity may be mediated by proteases or phospholipases rather than classical hemolysins (Vandenesch et al., 2012). It is also possible that homologous hemolysin genes or functional analogs were acquired through horizontal gene transfer (HGT) or retained for ecological adaptation. Unlike *S. aureus*, where hemolysins are key virulence factors, *S. xylosus* likely utilizes β-hemolysis for ecological fitness, particularly in food-associated environments such as plant surfaces. While this activity may enhance colonization potential, it does not necessarily indicate pathogenicity in healthy individuals, underscoring the phenotypic diversity and evolutionary plasticity of staphylococcal species (Eltwisy et al., 2020). The FFCShyA4 strain contained several putative resistance genes linked to resistance to lincosamide (1), tetracycline (4), fosfomycin (1), fluoroquinolone (16), macrolide (3), mupirocin (1), and salicylic acid (1) antimicrobials (Supplementary Figure S7). The antibiotic resistance profile of FFCShyA4 was compared with *S. aureus* ATCC1026 and *S. aureus* ATCC43300, highlighting key differences in their susceptibility patterns (Figure 5). FFCShyA4 shows resistance to several key antibiotics, particularly TE30 (tetracycline), MET5 (methicillin), CXM30 (cefuroxime), VA30 (Vancomycin), AX25 (amoxycillin), AM10 (ampicillin), OX1 (oxacillin), and LZD30 (linezolid), while displaying intermediate resistance to CIP5 (ciprofloxacin), C30 (chloramphenicol), and DA2 (clindamycin) (Supplementary Table S9). The MAR index was 0.67 for FFCShyA4, 0.70 for *S. aureus* ATCC 1026, and 0.62 for *S. aureus* ATCC 43300. Typically, a MAR index above 0.2 is considered significant, as it suggests exposure to antimicrobials in environments where misuse or overuse occurs, such as hospitals or livestock settings (Tang et al., 2023). The high MAR index and resistance to critical antibiotics, including methicillin and vancomycin, reflect possible exposure to antimicrobials in agricultural or post-harvest settings, raising concerns over antimicrobial misuse beyond clinical boundaries (Iwu et al., 2020). From a One Health perspective, the detection of multidrug-resistant *S. xylosus* on plant-based food products underscores the interconnectedness of human, animal, and environmental health, emphasizing the need for integrated surveillance of AMR across non-clinical reservoirs such as agricultural commodities, food supply chains, and market environments (Robinson et al., 2016). Compared to the reference *S. aureus* strains, FFCShyA4 exhibited phenotypic resistance to tetracycline, corroborating the resistance determinants identified through genomic analysis. The EggNOG annotation of FFShyA4 revealed the presence of *tpi*A and *bla* genes, indicating its potential for methicillin and β-lactam resistance, aligning with the observed multidrug resistance in the antibiogram. The *bla* gene suggests β-lactamase production, leading to resistance to penicillin and related antibiotics. Although the FFCShyA4 strain exhibited phenotypic resistance to methicillin, complementary PCR analysis using *mec*A and *mec*C primers failed to detect these genes. This discrepancy may be attributed to primer mismatches arising from sequence variations or the genomic location of *mec*A, potentially affecting primer binding efficiency. Besides, the presence of *tpi*A confirms the species identity, as it is a conserved housekeeping gene in *S. xylosus*. Early investigations into methicillin-resistant coagulase-negative staphylococci revealed that resistance to most β-lactam antibiotics arises primarily from the acquisition of the *mec*A or *mec*C genes, which encode alternative penicillin-binding proteins (PBPs) with reduced β-lactam binding affinity (Schwarz et al., 2018). The absence of *mec*A/*mec*C genes, common in livestock-associated *Staphylococcus*, does not exclude an animal origin but may indicate a less direct or recent exposure. Besides, the multidrug efflux pumps such as NorA which was annotated in the FFCShyA4 genome may contribute to methicillin resistance by actively exporting β-lactam antibiotics, reducing their intracellular concentration (Dashtbani-Roozbehani and Brown, 2021). Further comparative genomic and epidemiological studies would be necessary to confirm the specific origin of this strain. Another common resistance mechanism against penicillin involves enzymatic hydrolysis of the antibiotic by β-lactamases encoded by the *bla*Z or *bla*ARL genes (Schwarz et al., 2018). The absence of *mecA/mecC* alongside the detection of alternative resistance determinants (*bla*, *NorA*) supports the idea that non-traditional or environmental pathways may be driving resistance evolution in underregulated sectors, such as local markets. Early studies indicate that antimicrobial resistance in *Staphylococcus* spp. isolated from various food products varies significantly depending on the species and source of isolation (Resch et al., 2008). Among *S. xylosus* strains, resistance rates ranged from 22% for tetracycline to 69% for penicillin, with 93% of isolates originating from meat starter cultures. Notably, all coagulase-negative staphylococci (CNS) strains were fully susceptible to clinically relevant antibiotics such as chloramphenicol, clindamycin, cotrimoxazole, gentamicin, kanamycin, linezolid, neomycin, streptomycin, and vancomycin (Resch et al., 2008). Additionally, *S. xylosus* strains isolated from fermented soybean products were reported to be susceptible to chloramphenicol, erythromycin, gentamicin, kanamycin, lincomycin, oxacillin, tetracycline, and trimethoprim (Kong et al., 2022). However, 23 strains exhibited acquired phenotypic resistance to erythromycin, lincomycin, and tetracycline. Minimum inhibitory concentration (MIC) testing showed continuous or unimodal distribution patterns for these antibiotics, which did not align with the resistance profiles, suggesting possible discrepancies. These findings indicate that the current CLSI breakpoint values for ampicillin and penicillin G in *S. xylosus* may need to be re-evaluated. Moreover, the presence of acquired resistance to erythromycin, lincomycin, and tetracycline underscores the importance of performing antibiotic susceptibility testing before utilizing these strains in food-related applications (Kong et al., 2022). These observations underscore the necessity for coordinated, cross-sectoral surveillance strategies encompassing food production and environmental interfaces to monitor the emergence and dissemination of antimicrobial resistance, particularly in regions with insufficient regulation of antimicrobials use in agri-food systems.

## 4 Conclusion

This study highlights a multidrug-resistant *S. xylosus* strain FFCShyA4 from avocados as a potential foodborne threat, with genomic and phenotypic features suggesting cross-domain antimicrobial resistance and adaptation, reinforcing the need for integrated One Health surveillance in food systems. Genomic evidence suggests an animal-associated origin, potentially resulting from cross-contamination via human handling, market environments, or contact with animal-derived materials. The genome analysis reveals extensive structural rearrangements, and a wide array of genes linked to antibiotic resistance, virulence, and environmental adaptation. However, resistance genes (e.g., *fusA*, *pbp4*, *ileS*, *bla*, and *tpiA*), stress response elements, quorum sensing systems, and bacteriocin clusters, together with MGEs, indicate high potential for horizontal gene transfer and adaptation across niches. These features underscore the role of mobile produce surfaces as reservoirs and potential vectors for resistance genes, bridging ecosystems across the food production and consumption continuum. *In vitro* assays further confirmed hemolytic activity and resistance to multiple antimicrobial classes, highlighting its potential as an opportunistic pathogen. These findings highlight the critical need to extend antimicrobial resistance surveillance beyond clinical and veterinary contexts to include plant-derived food matrices. The comprehensive genomic analysis of FFCShyA4 illustrates how a single, well-characterized foodborne isolate can provide valuable insights into resistance mechanisms and their potential transmission across ecological boundaries. As such, this case underscores the importance of incorporating microbial genomics into food safety and public health strategies. Integrating such data into One Health surveillance systems is essential to guide effective interventions and evidence-based policymaking.

## Data Availability

The genome assembly data of *S. xylosus* FFCShyA4 are publicly available. This data can be found here: NCBI Sequence Read Archive, BioProject ID PRJNA1249250, BioSample accession SAMN47884644.

## Footnotes

^1^ https://beta.proksee.ca/projects
