# Supplementary material for: Genomic characterization of a multidrug-resistant Staphylococcus xylosus from Ecuadorian open market avocados: food safety and public health implications
Source: Front Microbiol. 2025 Jul 31;16:1629139. doi: 10.3389/fmicb.2025.1629139 (PMC12350326; doi:10.3389/fmicb.2025.1629139)
Supplement: Supplementary file 1 [file Table_1.docx]

Supplementary Material

Genomic Characterization of a Multidrug-Resistant *Staphylococcus xylosus* from Ecuadorian Open Market Avocados: Food Safety and Public Health Implications

Gabriela N. Tenea*, Evelyn Angamarca

Biofood and Nutraceutics Research and Development Group; Faculty of Engineering in Agricultural and Environmental Sciences, Universidad Técnica del Norte. Av. 17 de Julio s-21. Barrio El Olivo, Postcode: 100150, Ibarra, Ecuador

***Correspondence:**

Gabriela N. Tenea

gntenea@utn.edu.ec

**Table S1.** List of antibiotic tests and profile interpretation according to (A). CLSI (Clinical and Laboratory Standards Institute,2021); (B). EUCAST (European Committee on Antimicrobial Susceptibility Testing, 2021)

(A).

| **Class** | **Antibiotic name** | **Code** | **Susceptible (S)** | **Intermediate (I)** | **Resistant (R)** |
| --- | --- | --- | --- | --- | --- |
| Penicillin | Oxacillin | 0X1 | ≥18 | - | ≤17 |
|  | Ampicillin | AM10 | ≥17 | 14-16 | ≤13 |
|  | Methicillin | MET5 | ≥22 | - | ≤21 |
|  | Amoxicillin | AX25 | ≥17 | 14-16 | ≤13 |
|  | Penicillin G | P10 | ≥29 | - | ≤28 |
| Folate pathway antagonists | Sulphathiazole trimethoprim | STX25 | ≥16 | 11-15 | ≤10 |
| Annamycin | Rifampicin | RD5 | ≥20 | 17-19 | ≤16 |
| Fluoroquinolones | Ciprofloxacin | CIP5 | ≥21 | 16-20 | ≤15 |
| Phenicol | Chloramphenicol | C30 | ≥18 | 13-17 | ≤12 |
| Macrolide | Azithromycin | AZM15 | ≥18 | 14-17 | ≤13 |
|  | Erythromycin | E15 | ≥23 | 14-22 | ≤13 |
| Tetracyclines | Tetracycline | TE30 | ≥19 | 15-18 | ≤14 |
| Lincosamides | Clindamycin | DA2 | ≥21 | 15-20 | ≤14 |
| Aminoglycosides | Kanamycin | K30 | ≥18 | 14-17 | ≤13 |
|  | Gentamicin | CN10 | ≥15 | 13-14 | ≤12 |
|  | Amikacin | AN30 | ≥17 | 15–16 | ≤14 |
| Oxazolidinones | Linezolid | LZD30 | ≥21 | - | ≤20 |
| Glycopeptide | Vancomycin | VA30 | ≥17 | 15-16 | ≤14 |
| Aminocoumarin | Novobiocin | NV30 | ≥16 | - | ≤16 |
| Polypeptide | Bacitracin | B10 | ≥13 | - | - |
|  | Bacitracin | B0.04 | ≥10 | - | - |
| Cephalosporin | Ceftazidime | CAZ30 | ≥18 | 15-17 | ≤14 |
|  | Cefuroxime | CXM30 | ≥18 | 15-17 | ≤14 |
|  | Cefotaxime | CTX30 | ≥23 | 15-22 | ≤14 |

**(B).**

| **Class** | **Antibiotic name** | **Code** | **S** | **R** |
| --- | --- | --- | --- | --- |
| Penicillin | Oxacillin | 0X1 | - | - |
|  | Ampicillin | AM10 | - | - |
|  | Methicillin | MET5 | - | - |
|  | Amoxicillin | AX25 | - | - |
|  | Penicillin G | P10 | - | - |
| Folate pathway antagonists | Sulfamethoxazole trimethoprim | STX25 | ≥17 | <14 |
| Annamycin | Rifampicin | RD5 | ≥26 | <26 |
| Fluoroquinolones | Ciprofloxacin | CIP5 | ≥ 50 | <17 |
| Phenicol | Chloramphenicol | C30 | - | - |
| Macrolide | Azithromycin | AZM15 | - | - |
|  | Erythromycin | E15 | ≥21 | <21 |
| Tetracyclines | Tetracycline | TE30 | ≥22 | <22 |
| Lacosamide | Clindamycin | DA2 | ≥22 | <22 |
| Aminoglycosides | Kanamycin | K30 | - | - |
|  | Gentamicin | CN10 | ≥18 | <18 |
|  | Amikacin | AN30 | ≥15 | <15 |
| Oxazolidinones | Linezolid | LZD30 | ≥21 | <21 |
| Glycopeptide | Vancomycin | VA30 | - | - |
| Aminocoumarin | Novobiocin | NV30 | - | - |
| Polypeptide | Bacitracin | B10 | - | - |
|  | Bacitracin | B0.04 | - | - |
| Cephalosporin | Ceftazidime | CAZ30 | - | - |
|  | Cefuroxime | CXM30 | - | - |
|  | Cefotaxime | CTX30 | - | - |

**Table S2.** Primers and PCR conditions for the antibiotic and virulence genes teste in this study

| **Gene Name** | **Forward Primer** | **Reverse Primer** | **Amplicon weight (bp)** | **Conditions (Reference)** |
| --- | --- | --- | --- | --- |
| *hlg* (hemolysin) | 5’-GCCAATCCGTTATTAGAAAATGC-3’ | 5’-CCATAGACGTAGCAACGGAT-3’ | 937 | 32 cycles of denaturation at 95 °C for 1 min, annealing at 55 °C for hlg for 1 min, extension at 72 °C for 1 min, and a final extension at 72 °C for 2 min (Kumar et al., 2010). |
| *sdr*E (putative adhesin) | 5’-AGTAAAATGTGTCAAAAGA-3’ | 5’-TTGACTACCAGGCTATATC -3’ | 767 | 32 cycles of denaturation at 95 °C for 1 min, annealing at 45 °C for sdrE for 1 min, extension at 72 °C for 1 min, and a final extension at 72 °C for 2 min (Kumar et al., 2010) |
| *ica*A (intracellular adhesin) | 5’-GATTATGTAATGTGCTTGGA-3’ | 5’-ACTACTGCTGCGTTAATAAT-3’ | 770 | 35 cycles of denaturation at 95 °C for 1 min, annealing at 50 °C for 1 min, extension at 72 °C for 1 min, and a final extension at 72 °C for 2 min (Kumar et al., 2010) |
| *nuc* (thermonuclease) | 5’-GCGATTGATGGTGATACGGTT-3’ | 5’-AGCCAAGCCTTGACGAACTAAAGC-3’ | 270 | 35 cycles initial denaturation at 94°C for 5 min, followed by 35 cycles of 94°C for 1 min, 55°C for 30 s, and 72°C for 1 min. and final extension at 72°C for 7 min (Javid et al., 2018) |
| *mecA (methicillin gene A,* which encodes the altered penicillin-binding protein PBP-2a) | 5’-GTGAAGATATACCAAGTGATT-3’ | 5’-ATGCGCTATAGATTGAAAGGAT-3’ | 147 | 35 cycles, initial denaturation at 95°C for 15 min, followed by 10 cycles of 95°C for 45 s, 67°C for 45 s, and 72°C for 1.5 minutes; 25 cycles of 95°C for 45 s, 57°C for 45 s, and 72°C for 1.5 minutes; and extension at 72°C for 10 min. (Zhang et al., 2005) |
| *mecC (methicillin gene C,* a homologue of mecA) | 5’-GAAAAAAAGGCTTAGAACGCCTC-3’ | 5’-GAAGATCTTTTCCGTTTTCAGC-3’ | 138 | 35 cycles, initial denaturation at 95°C for 15 min, followed by 10 cycles of 95°C for 45 s, 67°C for 45 s, and 72°C for 1.5 minutes; 25 cycles of 95°C for 45 s, 57°C for 45 s, and 72°C for 1.5 minutes; and extension at 72°C for 10 min. (Zhang et al., 2005) |

**Table S3.** Genome features of *S. xylosus* FFCShyA4

| **Features** | **Values** |
| --- | --- |
| Genome length (bp) | 2,856,035 |
| Plasmids | 1 |
| GC content (%) | 32.61 |
| Total number of genes | 2720 |
| Coding genes | 2665 |
| tRNA number of assembled genome | 47 |
| rRNA number of assembled genome | 7 |
| tmRNA number of assembled genome | 1 |
| CRISPR-Cas array* | 2 |
| Prophage (intact region)** | 3 |
| Antibiotic acquired genes*** | None |
| Pathogenicity**** | Human pathogen |

*CRISPRFinder (<https://crisprcas.i2bc.paris-saclay.fr/CrisprCasFinder/Index>); **PHAge Search Tool Enhanced Release (PHASTER) ([http://phaster.ca](http://phaster.ca/)); ***ResFinder 4.1 (<https://cge.cbs.dtu.dk/services/ResFinder/>); ****PathogenFinder (<http://cge.cbs.dtu.dk/services/PathogenFinder/>).

**Table S4.** Top five genomes resulted by ANI analysis

| **Ranking** | **Similar genome** | **ANI (%)** | **Aln. Cov.** |
| --- | --- | --- | --- |
| **1** | *GCF_007992815.1_S.xylosus_NBRC_109770* | 99.97 | 94.19 |
| **2** | *GCF_925279085.1_S.xylosus_* | 99.95 | 94.44 |
| **3** | *GCF_019148995.1_S.xylosus_K19* | 99.95 | 94.64 |
| **4** | *GCF_024621885.1_S.xylosus_DSM_28566 99.94 94.35* | 99.94 | 94.35 |
| **5** | *GCF_019334165.1_S.xylosus_K46 99.92 94.45* | 99.92 | 94.45 |

Ranking : The order of the highest ANI value; Similar genome : Species name which has high ANI value.

ANI : % of relatedness at whole-genome level; Aln. Cov. : Alignment coverage. % of coverage by assembly sequence alignments against compared genome.

**Table S5.**  Summary of complete prophage regions identified on the FFCShyA4 genome using the PHASTER webserver

| Contig | Region | Region length | Completeness | Score | # Total proteins | Region position | Most common Phage | GC% |
| --- | --- | --- | --- | --- | --- | --- | --- | --- |
| 1 | 1 | 57.3 kb | Intact | 120 | 66 | 662858-720207 | PHAGE_Staphy 47_NC_007054(12) | 33.59 |

Region: the number assigned to the region; region length: the length of the sequence in that region; completeness: a prediction of whether the region contains a intact prophage based on the criteria: intact (score > 90%); score: the score of the region based on the mentioned criteria; # total proteins: the number of ORFs present in the region; Region position: the start and end positions of the region on the bacterial chromosome; Most common phage: the phages (s) with the highest number of proteins most similar to those in the region; GC%: the percentage of GC nucleotides of the region.

**Table S6**. Genomic Islands predicted in the FFCShyA4 genome

| Island start | Island end | Length | Gene ID | Locus | Gene start | Gene end | Strand | Product |
| --- | --- | --- | --- | --- | --- | --- | --- | --- |
| 643733 | 654372 | 10639 | glmS | FFCShyA4_02541 | 643733 | 645538 | 1 | glutamine--fructose-6-phosphate transaminase (isomerizing) |
| 643733 | 654372 | 10639 |  | FFCShyA4_02540 | 646643 | 647845 | 1 | hypothetical protein |
| 643733 | 654372 | 10639 |  | FFCShyA4_02539 | 647856 | 650981 | 1 | hypothetical protein |
| 643733 | 654372 | 10639 |  | FFCShyA4_02538 | 651042 | 651407 | 1 | hypothetical protein |
| 643733 | 654372 | 10639 |  | FFCShyA4_02537 | 651431 | 651775 | 1 | IS200/IS605 family transposase ISStin10 |
| 643733 | 654372 | 10639 |  | FFCShyA4_02536 | 652269 | 652625 | 1 | hypothetical protein |
| 643733 | 654372 | 10639 |  | FFCShyA4_02535 | 652667 | 653449 | 1 | ABC transporter ATP-binding protein |
| 643733 | 654372 | 10639 |  | FFCShyA4_02534 | 653518 | 654372 | 1 | HAD family hydrolase |
| 724777 | 741804 | 17027 | mazE | FFCShyA4_02458 | 724777 | 724947 | 1 | type II toxin-antitoxin system antitoxin MazE |
| 724777 | 741804 | 17027 |  | FFCShyA4_02457 | 724944 | 725306 | 1 | type II toxin-antitoxin system PemK/MazF family toxin |
| 724777 | 741804 | 17027 |  | FFCShyA4_02456 | 725655 | 726656 | 1 | PP2C family protein-serine/threonine phosphatase |
| 724777 | 741804 | 17027 |  | FFCShyA4_02455 | 726736 | 727062 | 1 | anti-sigma factor antagonist |
| 724777 | 741804 | 17027 | rsbW | FFCShyA4_02454 | 727064 | 727546 | 1 | anti-sigma B factor RsbW |
| 724777 | 741804 | 17027 | sigB | FFCShyA4_02453 | 727521 | 728291 | 1 | RNA polymerase sigma factor SigB |
| 724777 | 741804 | 17027 |  | FFCShyA4_02452 | 728438 | 730588 | 1 | Tex family protein |
| 724777 | 741804 | 17027 |  | FFCShyA4_02451 | 730581 | 730805 | 1 | SprT family protein |
| 724777 | 741804 | 17027 |  | FFCShyA4_02450 | 730957 | 731343 | 1 | hypothetical protein |
| 724777 | 741804 | 17027 |  | FFCShyA4_02449 | 731444 | 732493 | 1 | hypothetical protein |
| 724777 | 741804 | 17027 |  | FFCShyA4_02448 | 732490 | 734277 | 1 | hypothetical protein |
| 724777 | 741804 | 17027 |  | FFCShyA4_02447 | 734308 | 734769 | 1 | hypothetical protein |
| 724777 | 741804 | 17027 |  | FFCShyA4_02446 | 734969 | 735904 | 1 | hypothetical protein |
| 724777 | 741804 | 17027 |  | FFCShyA4_02445 | 736089 | 736673 | -1 | hypothetical protein |
| 724777 | 741804 | 17027 |  | FFCShyA4_02444 | 736692 | 738371 | -1 | hypothetical protein |
| 724777 | 741804 | 17027 |  | FFCShyA4_02443 | 738377 | 740719 | -1 | hypothetical protein |
| 724777 | 741804 | 17027 |  | FFCShyA4_02442 | 740731 | 741804 | -1 | hypothetical protein |
| 1533468 | 1577793 | 44325 |  | FFCShyA4_00654 | 1533468 | 1533662 | -1 | hypothetical protein |
| 1533468 | 1577793 | 44325 |  | FFCShyA4_00655 | 1534228 | 1534563 | 1 | hypothetical protein |
| 1533468 | 1577793 | 44325 |  | FFCShyA4_00656 | 1534582 | 1534761 | -1 | hypothetical protein |
| 1533468 | 1577793 | 44325 |  | FFCShyA4_00657 | 1534772 | 1535182 | -1 | YolD-like family protein |
| 1533468 | 1577793 | 44325 |  | FFCShyA4_00658 | 1535302 | 1535481 | 1 | hypothetical protein |
| 1533468 | 1577793 | 44325 |  | FFCShyA4_00659 | 1536356 | 1537117 | -1 | CHAP domain-containing protein |
| 1533468 | 1577793 | 44325 |  | FFCShyA4_00660 | 1537172 | 1538050 | -1 | hypothetical protein |
| 1533468 | 1577793 | 44325 |  | FFCShyA4_00661 | 1538109 | 1538384 | -1 | Holin |
| 1533468 | 1577793 | 44325 |  | FFCShyA4_00662 | 1538586 | 1538951 | -1 | hypothetical protein |
| 1533468 | 1577793 | 44325 |  | FFCShyA4_00663 | 1539019 | 1539180 | -1 | hypothetical protein |
| 1533468 | 1577793 | 44325 |  | FFCShyA4_00664 | 1539170 | 1540012 | -1 | hypothetical protein |
| 1533468 | 1577793 | 44325 |  | FFCShyA4_00665 | 1540057 | 1540461 | -1 | hypothetical protein |
| 1533468 | 1577793 | 44325 |  | FFCShyA4_00666 | 1540472 | 1541710 | -1 | hypothetical protein |
| 1533468 | 1577793 | 44325 |  | FFCShyA4_00667 | 1541722 | 1543383 | -1 | hypothetical protein |
| 1533468 | 1577793 | 44325 |  | FFCShyA4_00668 | 1543380 | 1543742 | -1 | hypothetical protein |
| 1533468 | 1577793 | 44325 |  | FFCShyA4_00669 | 1543735 | 1545324 | -1 | prophage endopeptidase tail family protein |
| 1533468 | 1577793 | 44325 |  | FFCShyA4_00670 | 1545334 | 1546197 | -1 | phage tail family protein |
| 1533468 | 1577793 | 44325 |  | FFCShyA4_00671 | 1546200 | 1552373 | -1 | phage tail tape measure protein |
| 1533468 | 1577793 | 44325 |  | FFCShyA4_00672 | 1552591 | 1552920 | -1 | hypothetical protein |
| 1533468 | 1577793 | 44325 |  | FFCShyA4_00673 | 1552984 | 1553451 | -1 | Ig-like domain-containing protein |
| 1533468 | 1577793 | 44325 |  | FFCShyA4_00674 | 1553546 | 1554133 | -1 | phage tail protein |
| 1533468 | 1577793 | 44325 |  | FFCShyA4_00675 | 1554173 | 1554568 | -1 | hypothetical protein |
| 1533468 | 1577793 | 44325 |  | FFCShyA4_00676 | 1554565 | 1554972 | -1 | hypothetical protein |
| 1533468 | 1577793 | 44325 |  | FFCShyA4_00677 | 1554972 | 1555325 | -1 | hypothetical protein |
| 1533468 | 1577793 | 44325 |  | FFCShyA4_00678 | 1555309 | 1555605 | -1 | hypothetical protein |
| 1533468 | 1577793 | 44325 |  | FFCShyA4_00679 | 1555678 | 1556802 | -1 | hypothetical protein |
| 1533468 | 1577793 | 44325 | clpP_1 | FFCShyA4_00680 | 1556829 | 1557569 | -1 | ATP-dependent Clp protease proteolytic subunit |
| 1533468 | 1577793 | 44325 |  | FFCShyA4_00681 | 1557566 | 1558753 | -1 | hypothetical protein |
| 1533468 | 1577793 | 44325 |  | FFCShyA4_00682 | 1558768 | 1560408 | -1 | terminase large subunit |
| 1533468 | 1577793 | 44325 |  | FFCShyA4_00683 | 1560405 | 1560707 | -1 | hypothetical protein |
| 1533468 | 1577793 | 44325 |  | FFCShyA4_00684 | 1560860 | 1561198 | -1 | hypothetical protein |
| 1533468 | 1577793 | 44325 |  | FFCShyA4_00685 | 1561532 | 1561939 | -1 | hypothetical protein |
| 1533468 | 1577793 | 44325 |  | FFCShyA4_00686 | 1561954 | 1563312 | -1 | hypothetical protein |
| 1533468 | 1577793 | 44325 |  | FFCShyA4_00687 | 1563302 | 1563592 | -1 | hypothetical protein |
| 1533468 | 1577793 | 44325 |  | FFCShyA4_00688 | 1563952 | 1566411 | -1 | hypothetical protein |
| 1533468 | 1577793 | 44325 |  | FFCShyA4_00689 | 1566500 | 1566646 | -1 | hypothetical protein |
| 1533468 | 1577793 | 44325 |  | FFCShyA4_00690 | 1566646 | 1566777 | -1 | transcriptional regulator |
| 1533468 | 1577793 | 44325 |  | FFCShyA4_00691 | 1566774 | 1567010 | -1 | hypothetical protein |
| 1533468 | 1577793 | 44325 |  | FFCShyA4_00692 | 1567049 | 1567360 | -1 | MazG-like family protein |
| 1533468 | 1577793 | 44325 |  | FFCShyA4_00693 | 1567361 | 1567915 | -1 | hypothetical protein |
| 1533468 | 1577793 | 44325 |  | FFCShyA4_00694 | 1567916 | 1568020 | -1 | hypothetical protein |
| 1533468 | 1577793 | 44325 |  | FFCShyA4_00695 | 1568021 | 1568230 | -1 | hypothetical protein |
| 1533468 | 1577793 | 44325 |  | FFCShyA4_00696 | 1568220 | 1568513 | -1 | hypothetical protein |
| 1533468 | 1577793 | 44325 |  | FFCShyA4_00697 | 1568514 | 1568753 | -1 | hypothetical protein |
| 1533468 | 1577793 | 44325 |  | FFCShyA4_00698 | 1568956 | 1569333 | -1 | hypothetical protein |
| 1533468 | 1577793 | 44325 |  | FFCShyA4_00699 | 1569317 | 1569526 | -1 | hypothetical protein |
| 1533468 | 1577793 | 44325 |  | FFCShyA4_00700 | 1569523 | 1569828 | -1 | hypothetical protein |
| 1533468 | 1577793 | 44325 |  | FFCShyA4_00701 | 1569834 | 1570391 | -1 | hypothetical protein |
| 1533468 | 1577793 | 44325 |  | FFCShyA4_00702 | 1570392 | 1570766 | -1 | hypothetical protein |
| 1533468 | 1577793 | 44325 |  | FFCShyA4_00703 | 1570780 | 1570968 | -1 | hypothetical protein |
| 1533468 | 1577793 | 44325 |  | FFCShyA4_00704 | 1570978 | 1572930 | -1 | hypothetical protein |
| 1533468 | 1577793 | 44325 |  | FFCShyA4_00705 | 1572989 | 1573537 | -1 | hypothetical protein |
| 1533468 | 1577793 | 44325 |  | FFCShyA4_00706 | 1573561 | 1574727 | -1 | hypothetical protein |
| 1533468 | 1577793 | 44325 |  | FFCShyA4_00707 | 1574724 | 1575074 | -1 | hypothetical protein |
| 1533468 | 1577793 | 44325 |  | FFCShyA4_00708 | 1575142 | 1575321 | -1 | hypothetical protein |
| 1533468 | 1577793 | 44325 |  | FFCShyA4_00709 | 1575318 | 1575530 | -1 | hypothetical protein |
| 1533468 | 1577793 | 44325 |  | FFCShyA4_00710 | 1575543 | 1575806 | -1 | hypothetical protein |
| 1533468 | 1577793 | 44325 |  | FFCShyA4_00711 | 1575821 | 1576045 | -1 | hypothetical protein |
| 1533468 | 1577793 | 44325 |  | FFCShyA4_00712 | 1576226 | 1576579 | 1 | hypothetical protein |
| 1533468 | 1577793 | 44325 |  | FFCShyA4_00713 | 1576593 | 1577087 | 1 | hypothetical protein |
| 1533468 | 1577793 | 44325 |  | FFCShyA4_00714 | 1577176 | 1577793 | 1 | hypothetical protein |
| 2091994 | 2098049 | 6055 | mnhF2 | FFCShyA4_02163 | 2091695 | 2091997 | -1 | Na+/H+ antiporter Mnh2 subunit F |
| 2091994 | 2098049 | 6055 | mnhE2 | FFCShyA4_02164 | 2091994 | 2092476 | -1 | Na+/H+ antiporter Mnh2 subunit E |
| 2091994 | 2098049 | 6055 | mnhD2 | FFCShyA4_02165 | 2092476 | 2093978 | -1 | Na+/H+ antiporter Mnh2 subunit D |
| 2091994 | 2098049 | 6055 | mnhC2 | FFCShyA4_02166 | 2093968 | 2094315 | -1 | Na+/H+ antiporter Mnh2 subunit C |
| 2091994 | 2098049 | 6055 | mnhB2 | FFCShyA4_02167 | 2094312 | 2094737 | -1 | Na+/H+ antiporter Mnh2 subunit B |
| 2091994 | 2098049 | 6055 |  | FFCShyA4_02168 | 2094724 | 2097120 | -1 | hypothetical protein |
| 2091994 | 2098049 | 6055 |  | FFCShyA4_02169 | 2097144 | 2097695 | -1 | tyrosine-type recombinase/integrase |
| 2091994 | 2098049 | 6055 |  | FFCShyA4_02170 | 2097843 | 2098049 | 1 | hypothetical protein |
| 2639456 | 2687454 | 47998 |  | FFCShyA4_01679 | 2639456 | 2641132 | 1 | recombinase family protein |
| 2639456 | 2687454 | 47998 |  | FFCShyA4_01678 | 2641235 | 2641576 | 1 | SAUGI family uracil-DNA glycosylase inhibitor |
| 2639456 | 2687454 | 47998 |  | FFCShyA4_01677 | 2641578 | 2641679 | 1 | hypothetical protein |
| 2639456 | 2687454 | 47998 |  | FFCShyA4_01676 | 2641672 | 2641983 | 1 | hypothetical protein |
| 2639456 | 2687454 | 47998 |  | FFCShyA4_01675 | 2641999 | 2642499 | 1 | hypothetical protein |
| 2639456 | 2687454 | 47998 |  | FFCShyA4_01674 | 2642520 | 2642966 | 1 | hypothetical protein |
| 2639456 | 2687454 | 47998 |  | FFCShyA4_01673 | 2643136 | 2644008 | 1 | HTH domain-containing protein |
| 2639456 | 2687454 | 47998 |  | FFCShyA4_01672 | 2644060 | 2644233 | 1 | hypothetical protein |
| 2639456 | 2687454 | 47998 |  | FFCShyA4_01671 | 2644366 | 2644857 | 1 | hypothetical protein |
| 2639456 | 2687454 | 47998 |  | FFCShyA4_01670 | 2644996 | 2645598 | 1 | hypothetical protein |
| 2639456 | 2687454 | 47998 |  | FFCShyA4_01669 | 2645618 | 2646169 | 1 | hypothetical protein |
| 2639456 | 2687454 | 47998 | galU_1 | FFCShyA4_01668 | 2646464 | 2647327 | 1 | UTP--glucose-1-phosphate uridylyltransferase GalU |
| 2639456 | 2687454 | 47998 |  | FFCShyA4_01667 | 2647358 | 2650231 | 1 | hypothetical protein |
| 2639456 | 2687454 | 47998 |  | FFCShyA4_01666 | 2650520 | 2652523 | 1 | hypothetical protein |
| 2639456 | 2687454 | 47998 |  | FFCShyA4_01665 | 2652574 | 2652669 | 1 | hypothetical protein |
| 2639456 | 2687454 | 47998 |  | FFCShyA4_01664 | 2653206 | 2654435 | 1 | hypothetical protein |
| 2639456 | 2687454 | 47998 | srmB | FFCShyA4_01663 | 2654710 | 2657598 | -1 | ATP-dependent RNA helicase SrmB |
| 2639456 | 2687454 | 47998 | nudG | FFCShyA4_01662 | 2657595 | 2657987 | -1 | CTP pyrophosphohydrolase |
| 2639456 | 2687454 | 47998 |  | FFCShyA4_01661 | 2658263 | 2659993 | 1 | hypothetical protein |
| 2639456 | 2687454 | 47998 |  | FFCShyA4_01660 | 2659986 | 2660324 | 1 | hypothetical protein |
| 2639456 | 2687454 | 47998 |  | FFCShyA4_01659 | 2660811 | 2661797 | 1 | hypothetical protein |
| 2639456 | 2687454 | 47998 |  | FFCShyA4_01658 | 2662795 | 2663445 | 1 | hypothetical protein |
| 2639456 | 2687454 | 47998 |  | FFCShyA4_01657 | 2664663 | 2665529 | 1 | LysR family transcriptional regulator |
| 2639456 | 2687454 | 47998 | ifcA | FFCShyA4_01656 | 2665655 | 2667148 | 1 | Fumarate reductase flavoprotein subunit |
| 2639456 | 2687454 | 47998 |  | FFCShyA4_01655 | 2667506 | 2668486 | 1 | tripartite tricarboxylate transporter substrate binding protein |
| 2639456 | 2687454 | 47998 |  | FFCShyA4_01654 | 2668499 | 2668942 | 1 | hypothetical protein |
| 2639456 | 2687454 | 47998 |  | FFCShyA4_01653 | 2668957 | 2670456 | 1 | tripartite tricarboxylate transporter permease |
| 2639456 | 2687454 | 47998 |  | FFCShyA4_01652 | 2670794 | 2672443 | 1 | KsdD-like steroid dehydrogenase |
| 2639456 | 2687454 | 47998 | yiaC | FFCShyA4_01651 | 2672663 | 2673094 | 1 | Peptidyl-lysine N-acetyltransferase YiaC |
| 2639456 | 2687454 | 47998 |  | FFCShyA4_01650 | 2673294 | 2674625 | -1 | YjiH family protein |
| 2639456 | 2687454 | 47998 |  | FFCShyA4_01649 | 2674667 | 2675347 | -1 | flavin reductase family protein |
| 2639456 | 2687454 | 47998 |  | FFCShyA4_01648 | 2675787 | 2676176 | 1 | tautomerase family protein |
| 2639456 | 2687454 | 47998 |  | FFCShyA4_01647 | 2676309 | 2676623 | -1 | hypothetical protein |
| 2639456 | 2687454 | 47998 |  | FFCShyA4_01646 | 2676799 | 2677560 | 1 | SDR family NAD(P)-dependent oxidoreductase |
| 2639456 | 2687454 | 47998 |  | FFCShyA4_01645 | 2677902 | 2678219 | -1 | YSIRK-type signal peptide-containing protein |
| 2639456 | 2687454 | 47998 | lip | FFCShyA4_01644 | 2678373 | 2680061 | -1 | Lipase |
| 2639456 | 2687454 | 47998 |  | FFCShyA4_01643 | 2680509 | 2681558 | 1 | ABC transporter permease |
| 2639456 | 2687454 | 47998 |  | FFCShyA4_01642 | 2681576 | 2682244 | 1 | ABC transporter ATP-binding protein |
| 2639456 | 2687454 | 47998 |  | FFCShyA4_01641 | 2682385 | 2683065 | -1 | flavin reductase family protein |
| 2639456 | 2687454 | 47998 |  | FFCShyA4_01640 | 2683186 | 2684364 | -1 | MFS transporter |
| 2639456 | 2687454 | 47998 |  | FFCShyA4_01639 | 2684484 | 2684729 | 1 | hypothetical protein |
| 2639456 | 2687454 | 47998 |  | FFCShyA4_01638 | 2684926 | 2686425 | 1 | NAD-dependent epimerase/dehydratase family protein |
| 2639456 | 2687454 | 47998 |  | FFCShyA4_01637 | 2686476 | 2686976 | 1 | TspO/MBR family protein |
| 2639456 | 2687454 | 47998 |  | FFCShyA4_01636 | 2686981 | 2687454 | 1 | hypothetical protein |

**Table S7.** Virulence factors with more than 60% sequence similarity annotated in the FFCShyA4 genome

| Gene ID | Similarity (%) | Gene description |
| --- | --- | --- |
| gene00252 | 62.1 | VFG043573(gb\|NP_219906) (dnaK) chaperone protein DnaK |
| gene00270 | 62.7 | VFG009717(gb\|WP_011741135) (sigA/rpoV) RNA polymerase sigma factor |
| gene00322 | 66.7 | VFG048851(gb\|WP_015958700) (gndA) NADP-dependent phosphogluconate dehydrogenase |
| gene00503 | 68.2 | VFG037030(gb\|WP_002221625) (katA) catalase |
| gene00514 | 80.2 | VFG004728(gb\|WP_011303214) (nuc) thermonuclease family protein |
| gene00604 | 68.1 | VFG011430(gb\|WP_002963616) (acpXL) acyl carrier protein |
| gene00830 | 84.5 | VFG004478(gb\|WP_011303453) (atl) GW dipeptide domain-containing protein |
| gene00927 | 70.4 | VFG044272(gb\|WP_000974906) (NWMN_RS12030) iron ABC transporter permease |
| gene00928 | 61 | VFG044273(gb\|WP_001214661) (NWMN_RS12035) ABC transporter substrate-binding protein |
| gene00931 | 60.9 | VFG044276(gb\|WP_000170403) (sfaA) staphyloferrin A export MFS transporter |
| gene01006 | 61.6 | VFG016309(gb\|WP_001084641) (galE) UDP-glucose 4-epimerase GalE |
| gene01350 | 64.3 | VFG004775(gb\|WP_011302266) (sspA) serine protease; V8 protease; glutamyl endopeptidase |
| gene01373 | 60.3 | VFG006496(gb\|WP_011115254) (ureG) urease accessory protein (ureG) |
| gene01546 | 63.1 | VFG004542(gb\|WP_002497699) (icaA) N-acetylglucosaminyltransferase, involved in polysaccharide intercellular adhesin(PIA) synthesis |
| gene01549 | 60.8 | VFG004519(gb\|WP_000723844) (icaC) intercellular adhesion protein C, involved in polysaccharide intercellular adhesin(PIA) synthesis |
| gene01637 | 68.5 | VFG016433(gb\|WP_000757777) (galU) UTP--glucose-1-phosphate uridylyltransferase GalU |
| gene01866 | 66 | VFG037030(gb\|WP_002221625) (katA) catalase |
| gene01978 | 79.8 | VFG005579(gb\|WP_000022815) (eno) phosphopyruvate hydratase |
| gene01982 | 70.1 | VFG019077(gb\|WP_000260685) (plr/gapA) type I glyceraldehyde-3-phosphate dehydrogenase |
| gene01988 | 77.3 | VFG000077(gb\|NP_465991) (clpP) ATP-dependent Clp protease proteolytic subunit |
| gene02060 | 70.6 | VFG016433(gb\|WP_000757777) (galU) UTP--glucose-1-phosphate uridylyltransferase GalU |
| gene02265 | 74.2 | VFG046475(gb\|WP_013922406) (tufA) elongation factor Tu |
| gene02288 | 69.5 | VFG000079(gb\|NP_463763) (clpC) endopeptidase Clp ATP-binding chain C |
| gene02330 | 67 | VFG012102(gb\|WP_011967678) (groEL) chaperonin GroEL |
| gene02457 | 61.9 | VFG005130(gb\|WP_000723436) (cap8P) type 8 capsular polysaccharide synthesis protein Cap8P |
| gene02530 | 67.7 | VFG043453(gb\|WP_001829380) (aae) autolysin/adhesin Aae |
| gene02534 | 73.1 | VFG004765(gb\|WP_002437845) (lip) triacylglycerol lipase precursor |

**Table S8.** Description of the Cluster gene regions of FFCShyA4 detected showing similarity on the MIBig database

| **Contig/ location** | **Reference** | **Similarity score** | **Type** | **Compound(s)** | **Organism** |
| --- | --- | --- | --- | --- | --- |
| contig 1.1/ 190,818 - 211,636 nt. | [BGC0001839](https://mibig.secondarymetabolites.org/repository/BGC0001839/index.html#r1c1) | 0.31 | Terpene | squalestatin S1 | *Aspergillus sp. Z5* |
|  | [BGC0001685](https://mibig.secondarymetabolites.org/repository/BGC0001685/index.html#r1c1) | 0.18 | Other | N-acyloxyacyl lysine | *Raoultella ornithinolytica 10-5246* |
|  | [BGC0000919](https://mibig.secondarymetabolites.org/repository/BGC0000919/index.html#r1c1) | 0.16 | Other | phaseolotoxin | *Pseudomonas syringae* |
|  | [BGC0002382](https://mibig.secondarymetabolites.org/repository/BGC0002382/index.html#r1c1) | 0.1 | RiPP | grimoviridin | *Grimontia marina* |
|  | [BGC0000707](https://mibig.secondarymetabolites.org/repository/BGC0000707/index.html#r1c1) | 0.09 | Saccharide | kasugamycin | *Streptomyces kasugaensis* |
|  | [BGC0002148](https://mibig.secondarymetabolites.org/repository/BGC0002148/index.html#r1c1) | 0.08 | Alkaloid | guanitoxin | *Sphaerospermopsis torques-reginae ITEP-024* |
|  | [BGC0000905](https://mibig.secondarymetabolites.org/repository/BGC0000905/index.html#r1c1) | 0.08 | Other | heme D1 | *Heliobacillus mobilis* |
|  | [BGC0000906](https://mibig.secondarymetabolites.org/repository/BGC0000906/index.html#r1c1) | 0.08 | Other | heme D1 | *Heliophilum fasciatum* |
|  | [BGC0002039](https://mibig.secondarymetabolites.org/repository/BGC0002039/index.html#r1c1) | 0.07 | Other | coformycin, formycin A | *Nocardia interforma ATCC 21072* |
|  | [BGC0001363](https://mibig.secondarymetabolites.org/repository/BGC0001363/index.html#r1c1) | 0.07 | NRP, Polyketide | ralsolamycin | *Ralstonia solanacearum GMI1000* |
| contig 2.1 / 1 - 32,446 nt. | [BGC0000944](https://mibig.secondarymetabolites.org/repository/BGC0000944/index.html#r1c1) | 0.54 | Other (Non-NRP siderophore) | staphyloferrin A | *Staphylococcus aureus subsp. aureus NCTC 8325* |
|  | [BGC0001498](https://mibig.secondarymetabolites.org/repository/BGC0001498/index.html#r1c1) | 0.33 | Other | aerobactin | *Xenorhabdus szentirmaii DSM 16338* |
|  | [BGC0002073](https://mibig.secondarymetabolites.org/repository/BGC0002073/index.html#r1c1) | 0.25 | NRP | dehydroxynocardamine | *Corynebacterium propinquum* |
|  | [BGC0002683](https://mibig.secondarymetabolites.org/repository/BGC0002683/index.html#r1c1) | 0.24 | Other | schizokinen | *Nostoc sp. PCC 7120 = FACHB-418* |
|  | [BGC0002471](https://mibig.secondarymetabolites.org/repository/BGC0002471/index.html#r1c1) | 0.24 | Other | baumannoferrin A, baumannoferrin B | *Acinetobacter baumannii AYE* |
|  | [BGC0001870](https://mibig.secondarymetabolites.org/repository/BGC0001870/index.html#r1c1) | 0.24 | Other | putrebactin, avaroferrin | *Xenorhabdus budapestensis* |
|  | [BGC0000947](https://mibig.secondarymetabolites.org/repository/BGC0000947/index.html#r1c1) | 0.21 | Other | vibrioferrin | *Vibrio alginolyticus* |
|  | [BGC0002470](https://mibig.secondarymetabolites.org/repository/BGC0002470/index.html#r1c1) | 0.2 | Other | synechobactin C9, synechobactin C11, synechobactin 13, synechobactin 14, synechobactin 16, synechobactin A, synechobactin B, synechobactin C | *Synechococcus sp. PCC 7002* |
|  | [BGC0002682](https://mibig.secondarymetabolites.org/repository/BGC0002682/index.html#r1c1) | 0.2 | Other | aerobactin | *Vibrio mimicus* |
|  | [BGC0000939](https://mibig.secondarymetabolites.org/repository/BGC0000939/index.html#r1c1) | 0.18 | Other | aerobactin | *Grimontia hollisae* |
| contig 2.2 / 383,259 - 424,428 nt | [BGC0000286](https://mibig.secondarymetabolites.org/repository/BGC0000286/index.html#r1c1) | 0.23 | Polyketide | viguiepinol | *Streptomyces sp. KO-3988* |
|  | [BGC0002561](https://mibig.secondarymetabolites.org/repository/BGC0002561/index.html#r1c1) | 0.2 | Alkaloid | phenazine SA, phenazine SB, phenazine SC | *Streptomyces sp.* |
|  | [BGC0000867](https://mibig.secondarymetabolites.org/repository/BGC0000867/index.html#r1c1) | 0.17 | Other | polyhydroxyalkanoic acids | *Ectothiorhodospira shaposhnikovii* |
|  | [BGC0000866](https://mibig.secondarymetabolites.org/repository/BGC0000866/index.html#r1c1) | 0.17 | Other | polyhydroxyalkanoate | *Burkholderia sp. DSM 9242* |
|  | [BGC0000255](https://mibig.secondarymetabolites.org/repository/BGC0000255/index.html#r1c1) | 0.17 | Polyketide | pederin | *Uncultured bacterium* |
|  | [BGC0000922](https://mibig.secondarymetabolites.org/repository/BGC0000922/index.html#r1c1) | 0.17 | Other | pseudomonas quinolone signal (PQS) | *Pseudomonas aeruginosa PAO1* |
|  | [BGC0001739](https://mibig.secondarymetabolites.org/repository/BGC0001739/index.html#r1c1) | 0.16 | Other | phosphonoacetic Acid | *Streptomyces sp. NRRL F-525* |
|  | [BGC0002284](https://mibig.secondarymetabolites.org/repository/BGC0002284/index.html#r1c1) | 0.16 | Other | corynecin III, corynecin I, corynecin II | *Rhodococcus sp. H-CA8f* |
|  | [BGC0000205](https://mibig.secondarymetabolites.org/repository/BGC0000205/index.html#r1c1) | 0.16 | Polyketide | bryostatin | *Candidatus Endobugula sertula* |
|  | [BGC0000934](https://mibig.secondarymetabolites.org/repository/BGC0000934/index.html#r1c1) | 0.16 | Other (Phenazine) | 5-acetyl-5,10-dihydrophenazine-1-carboxylic acid, 5-(2-hydroxyacetyl)-5,10-dihydrophenazine-1-carboxylic acid, endophenazine A1, endophenazine F, endophenazine G | *Kitasatospora sp. HKI 714* |
| contig 2.3 / 467,422 - 514,564 nt. | [BGC0000308](https://mibig.secondarymetabolites.org/repository/BGC0000308/index.html#r1c1) | 0.48 | NRP | aureusimine A, aureusimine B, aureusimine C | *Staphylococcus aureus subsp. aureus str. JKD6008* |
|  | [BGC0000375](https://mibig.secondarymetabolites.org/repository/BGC0000375/index.html#r1c1) | 0.36 | NRP | indigoidine | *Streptomyces chromofuscus* |
|  | [BGC0001561](https://mibig.secondarymetabolites.org/repository/BGC0001561/index.html#r1c1) | 0.29 | NRP | curacomycin | *Streptomyces curacoi* |
|  | [BGC0000727](https://mibig.secondarymetabolites.org/repository/BGC0000727/index.html#r1c1) | 0.29 | Saccharide | indigoidine | *Streptomyces aureofaciens* |
|  | [BGC0002710](https://mibig.secondarymetabolites.org/repository/BGC0002710/index.html#r1c1) | 0.29 | NRP | metachelin C, metachelin A, metachelin A-CE, metachelin B, dimerumic acid 11-mannoside, dimerumic acid | *Metarhizium robertsii ARSEF 23* |
|  | [BGC0000783](https://mibig.secondarymetabolites.org/repository/BGC0000783/index.html#r1c1) | 0.27 | Saccharide | O-antigen | *Xanthomonas oryzae pv. oryzae* |
|  | [BGC0002656](https://mibig.secondarymetabolites.org/repository/BGC0002656/index.html#r1c1) | 0.26 | Polyketide | oryzanaphthopyran A, oryzanaphthopyran B, oryzanaphthopyran C, oryzanthrone A, oryzanthrone B, chlororyzanthrone A, chlororyzanthrone B | *Streptacidiphilus oryzae TH49* |
|  | [BGC0001132](https://mibig.secondarymetabolites.org/repository/BGC0001132/index.html#r1c1) | 0.24 | NRP | xenotetrapeptide | *Xenorhabdus nematophila ATCC 19061* |
|  | [BGC0001128](https://mibig.secondarymetabolites.org/repository/BGC0001128/index.html#r1c1) | 0.24 | NRP | gamexpeptide C | *Photorhabdus laumondii subsp. laumondii TTO1* |
|  | [BGC0002716](https://mibig.secondarymetabolites.org/repository/BGC0002716/index.html#r1c1) | 0.24 | NRP | gamexpeptide A, gamexpeptide B, gamexpeptide E, luminmide B, luminmide D, luminmide E, luminmide F, luminmide G | *Photorhabdus laumondii subsp. laumondii* |
| contig 3.1/ 117,369 - 155,761 nt | [BGC0002487](https://mibig.secondarymetabolites.org/repository/BGC0002487/index.html#r1c1) | 0.64 | Other | staphylopine | *Staphylococcus aureus subsp. aureus Mu50* |
|  | [BGC0002488](https://mibig.secondarymetabolites.org/repository/BGC0002488/index.html#r1c1) | 0.53 | Other | bacillopaline | *Paenibacillus mucilaginosus KNP414* |
|  | [BGC0002116](https://mibig.secondarymetabolites.org/repository/BGC0002116/index.html#r1c1) | 0.33 | RiPP | darobactin A | *Photorhabdus khanii HGB 1456* |
|  | [BGC0000554](https://mibig.secondarymetabolites.org/repository/BGC0000554/index.html#r1c1) | 0.32 | RiPP | SRO15-3108 | *Streptomyces filamentosus NRRL 15998* |
|  | [BGC0000500](https://mibig.secondarymetabolites.org/repository/BGC0000500/index.html#r1c1) | 0.32 | RiPP | carnolysin A1, carnolysin A2 | *Carnobacterium maltaromaticum* |
|  | [BGC0000647](https://mibig.secondarymetabolites.org/repository/BGC0000647/index.html#r1c1) | 0.32 | Terpene | carotenoid | *Rhodobacter sphaeroides* |
|  | [BGC0001210](https://mibig.secondarymetabolites.org/repository/BGC0001210/index.html#r1c1) | 0.31 | RiPP | pseudomycoicidin | *Bacillus pseudomycoides DSM 12442* |
|  | [BGC0002630](https://mibig.secondarymetabolites.org/repository/BGC0002630/index.html#r1c1) | 0.31 | RiPP | thatisin | *Lysobacter antibioticus* |
|  | [BGC0000590](https://mibig.secondarymetabolites.org/repository/BGC0000590/index.html#r1c1) | 0.31 | RiPP | microcin N | *Escherichia coli* |
|  | [BGC0000589](https://mibig.secondarymetabolites.org/repository/BGC0000589/index.html#r1c1) | 0.31 | RiPP | microcin M | *Escherichia coli Nissle 1917* |
| contig 3.2/ 248,931 - 293,307 nt | [BGC0000457](https://mibig.secondarymetabolites.org/repository/BGC0000457/index.html#r1c1) | 0.31 | NRP | vicibactin | *Rhizobium etli CFN 42* |
|  | [BGC0000343](https://mibig.secondarymetabolites.org/repository/BGC0000343/index.html#r1c1) | 0.26 | NRP | enterobactin | *Pseudomonas sp. J465* |
|  | [BGC0002287](https://mibig.secondarymetabolites.org/repository/BGC0002287/index.html#r1c1) | 0.26 | NRP | mutanocyclin, leuvalin, tyrvalin | *Streptococcus mutans* |
|  | [BGC0002058](https://mibig.secondarymetabolites.org/repository/BGC0002058/index.html#r1c1) | 0.26 | Polyketide, NRP | mutanocyclin | *Streptococcus mutans B04Sm5* |
|  | [BGC0000342](https://mibig.secondarymetabolites.org/repository/BGC0000342/index.html#r1c1) | 0.25 | NRP | enniatin | *Fusarium equiseti* |
|  | [BGC0002437](https://mibig.secondarymetabolites.org/repository/BGC0002437/index.html#r1c1) | 0.25 | NRP | thermoactinoamide A | *Thermoactinomyces sp. AS95* |
|  | [BGC0002135](https://mibig.secondarymetabolites.org/repository/BGC0002135/index.html#r1c1) | 0.24 | NRP | bovienimide A | *Xenorhabdus bovienii SS-2004* |
|  | [BGC0002518](https://mibig.secondarymetabolites.org/repository/BGC0002518/index.html#r1c1) | 0.24 | NRP | syringafactin A, syringafactin C | *Pseudomonas sp. SZ57* |
|  | [BGC0001863](https://mibig.secondarymetabolites.org/repository/BGC0001863/index.html#r1c1) | 0.24 | RiPP, Terpene | bacillicn CER074 | *Bacillus mycoides* |
|  | [BGC0001135](https://mibig.secondarymetabolites.org/repository/BGC0001135/index.html#r1c1) | 0.24 | NRP | bicornutin A1, bicornutin A2 | *Xenorhabdus budapestensis* |
| contig 3.3 / 365,978 - 422,774 nt | [BGC0000308](https://mibig.secondarymetabolites.org/repository/BGC0000308/index.html#r1c1) | 0.37 | NRP | aureusimine A, aureusimine B, aureusimine C | *Staphylococcus aureus subsp. aureus str. JKD6008* |
|  | [BGC0002135](https://mibig.secondarymetabolites.org/repository/BGC0002135/index.html#r1c1) | 0.32 | NRP | bovienimide A | *Xenorhabdus bovienii SS-2004* |
|  | [BGC0002518](https://mibig.secondarymetabolites.org/repository/BGC0002518/index.html#r1c1) | 0.32 | NRP | syringafactin A, syringafactin C | *Pseudomonas sp. SZ57* |
|  | [BGC0001686](https://mibig.secondarymetabolites.org/repository/BGC0001686/index.html#r1c1) | 0.3 | NRP | N-octanoyl-Met-Phe-H | *Clostridium sp. CAG:567* |
|  | [BGC0001135](https://mibig.secondarymetabolites.org/repository/BGC0001135/index.html#r1c1) | 0.3 | NRP | bicornutin A1, bicornutin A2 | *Xenorhabdus budapestensis* |
|  | [BGC0001844](https://mibig.secondarymetabolites.org/repository/BGC0001844/index.html#r1c1) | 0.29 | NRP | holrhizin | *Paraburkholderia rhizoxinica HKI 454* |
|  | [BGC0002437](https://mibig.secondarymetabolites.org/repository/BGC0002437/index.html#r1c1) | 0.29 | NRP | thermoactinoamide A | *Thermoactinomyces sp. AS95* |
|  | [BGC0001833](https://mibig.secondarymetabolites.org/repository/BGC0001833/index.html#r1c1) | 0.29 | NRP | icosalide A, icosalide B | *Burkholderia gladioli* |
|  | [BGC0002660](https://mibig.secondarymetabolites.org/repository/BGC0002660/index.html#r1c1) | 0.27 | NRP | pentacitidin A, pentacitidin B | *Chitinophaga eiseniae* |
|  | [BGC0001758](https://mibig.secondarymetabolites.org/repository/BGC0001758/index.html#r1c1) | 0.27 | NRP | rhizomide A, rhizomide B, rhizomide C | *Paraburkholderia rhizoxinica HKI 454* |
| contig 5.1 / 18,213 - 38,922 nt. | [BGC0002585](https://mibig.secondarymetabolites.org/repository/BGC0002585/index.html#r1c1) | 0.16 | Other | ubericin K | *Streptococcus uberis* |
|  | [BGC0002579](https://mibig.secondarymetabolites.org/repository/BGC0002579/index.html#r1c1) | 0.16 | RiPP | carnobacteriocin XY | *Carnobacterium maltaromaticum* |
|  | [BGC0001687](https://mibig.secondarymetabolites.org/repository/BGC0001687/index.html#r1c1) | 0.15 | Other | N-tetradecanoyl tyrosine | *uncultured bacterium CSL12* |
|  | [BGC0002667](https://mibig.secondarymetabolites.org/repository/BGC0002667/index.html#r1c1) | 0.14 | RiPP | estericin A | *Clostridium estertheticum* |
|  | [BGC0000540](https://mibig.secondarymetabolites.org/repository/BGC0000540/index.html#r1c1) | 0.13 | RiPP | paenibacillin | *Paenibacillus polymyxa OSY-DF* |
|  | [BGC0000624](https://mibig.secondarymetabolites.org/repository/BGC0000624/index.html#r1c1) | 0.09 | RiPP | salivaricin CRL1328 α peptide, salivaricin CRL1328 β peptide | *Lactobacillus salivarius* |
|  | [BGC0001872](https://mibig.secondarymetabolites.org/repository/BGC0001872/index.html#r1c1) | 0.08 | Polyketide | fabclavine-polyamine | *Xenorhabdus bovienii SS-2004* |
|  | [BGC0001053](https://mibig.secondarymetabolites.org/repository/BGC0001053/index.html#r1c1) | 0.08 | NRP, Polyketide | tubulysin A | *Archangium disciforme* |
|  | [BGC0001514](https://mibig.secondarymetabolites.org/repository/BGC0001514/index.html#r1c1) | 0.07 | Other | aristeromycin | *Streptomyces citricolor* |
|  | [BGC0001442](https://mibig.secondarymetabolites.org/repository/BGC0001442/index.html#r1c1) | 0.07 | NRP | actinonin | *Streptomyces sp. strain ATCC 14903* |
| contig 9.1 / 1 - 17,786 nt. | [BGC0000650](https://mibig.secondarymetabolites.org/repository/BGC0000650/index.html#r1c1) | 0.32 | Terpene | carotenoid | *Algoriphagus sp. KK10202C* |
|  | [BGC0000647](https://mibig.secondarymetabolites.org/repository/BGC0000647/index.html#r1c1) | 0.32 | Terpene | carotenoid | *Rhodobacter sphaeroides* |
|  | [BGC0000644](https://mibig.secondarymetabolites.org/repository/BGC0000644/index.html#r1c1) | 0.25 | Terpene | carotenoid | *Dietzia sp. CQ4* |
|  | [BGC0002674](https://mibig.secondarymetabolites.org/repository/BGC0002674/index.html#r1c1) | 0.24 | RiPP | enterocin F4-9 | *Enterococcus faecalis* |
|  | [BGC0000648](https://mibig.secondarymetabolites.org/repository/BGC0000648/index.html#r1c1) | 0.23 | Terpene | carotenoid | *Myxococcus xanthus* |
|  | [BGC0001864](https://mibig.secondarymetabolites.org/repository/BGC0001864/index.html#r1c1) | 0.23 | RiPP | listeriocytocin | *Listeria monocytogenes SLCC2540* |
|  | [BGC0000645](https://mibig.secondarymetabolites.org/repository/BGC0000645/index.html#r1c1) | 0.23 | Terpene | carotenoid | *Halobacillus halophilus DSM 2266* |
|  | [BGC0001909](https://mibig.secondarymetabolites.org/repository/BGC0001909/index.html#r1c1) | 0.21 | Polyketide | strobilurin A | *Strobilurus tenacellus* |
|  | [BGC0001456](https://mibig.secondarymetabolites.org/repository/BGC0001456/index.html#r1c1) | 0.21 | Terpene | isorenieratene | *Streptomyces argillaceus* |
|  | [BGC0000777](https://mibig.secondarymetabolites.org/repository/BGC0000777/index.html#r1c1) | 0.21 | Saccharide | lipopolysaccharide | *Escherichia coli* |

**Table S9**. Antibiotic profile of *S. xylosus* FFCShyA4 compared with *S. aureus* ATCC1026 and *S. aureus* ATCC43300.

| **Antibiotics** | **Zone of inhibition (mm)** | | |
| --- | --- | --- | --- |
|  | ***S. aureus* ATCC 1026** | ***S. aureus* ATCC 43300** | ***S. xylosus* FFCShyA4** |
| **TE30** | 26 | 26 | 19 |
| **CXM30** | 6 | 18 | 18 |
| **AM10** | 6 | 6 | 6 |
| **AX25** | 6 | 7 | 6 |
| **K30** | 6 | 6 | 12 |
| **CN10** | 6 | 6.9 | 7 |
| **VAN30** | 13 | 12.8 | 6 |
| **MET5** | 6 | 17.7 | 6 |
| **OX1** | 6 | 10.8 | 10,5 |
| **SXT25** | 14 | 21.5 | 18.5 |
| **RD5** | 29.3 | 24.8 | 12 |
| **CIP5** | 8.7 | 26.6 | 20 |
| **C30** | 20.5 | 19.5 | 24 |
| **AZM15** | 6 | 6 | 10 |
| **DA2** | 8.6 | 17.3 | 12 |
| **P10** | 6 | 6 | 15 |
| **LZD30** | 29.5 | 16 | 24 |
| **E15** | 6 | 8.8 | 15 |
| **NV30** | 29.4 | 27.4 | 11 |
| **AN30** | 14 | 13 | 19 |
| **CTX30** | 6 | 15 | 20 |
| **CAZ30** | 6 | 10 | 11 |
| **B10** | 14 | 15 | 17 |
| **B0.04** | 6 | 6 | 8 |
| MAR*  % | 0.708 | 0.625 | 0.667 |

* MAR index is calculated as the ratio between the number of antibiotics that an isolate is resistant to and the total number of antibiotics the organism is exposed to. A MAR greater than 0.2 means that the high risk source of contamination is where antibiotics are frequently used; Colors: Red: resistant; blue: intermediate resistant; Green: sensible

**Figure S1**. Percent identity heatmap (A) and alignment coverage heatmap (B) resulted by ANI analysis

(A).


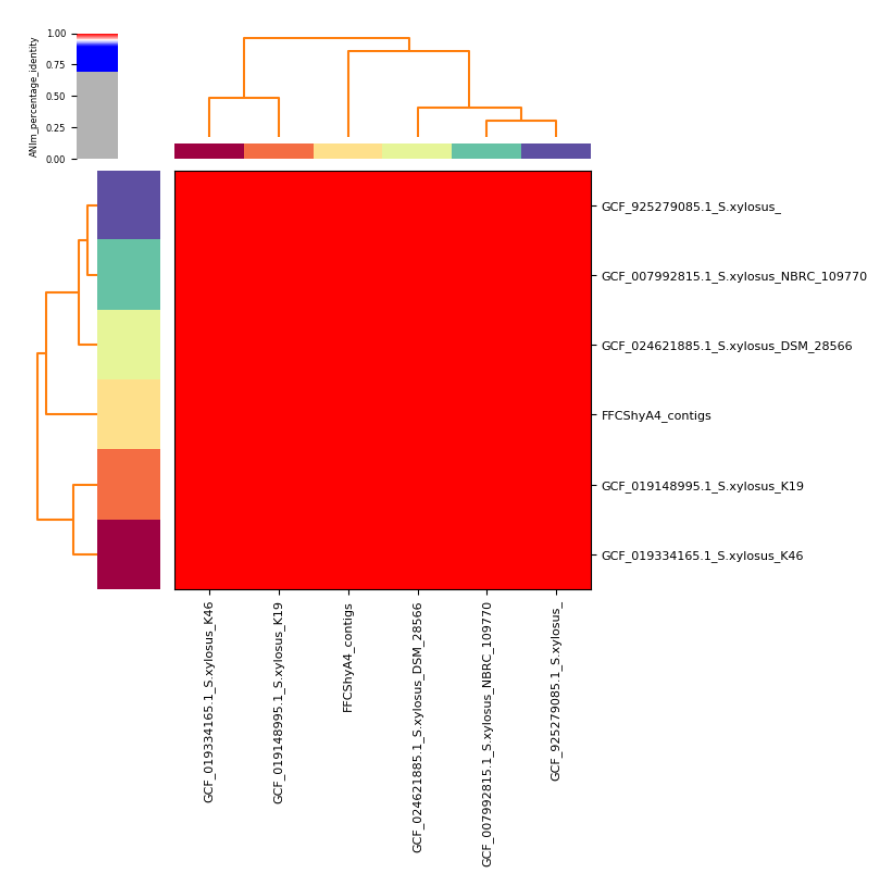


(B).
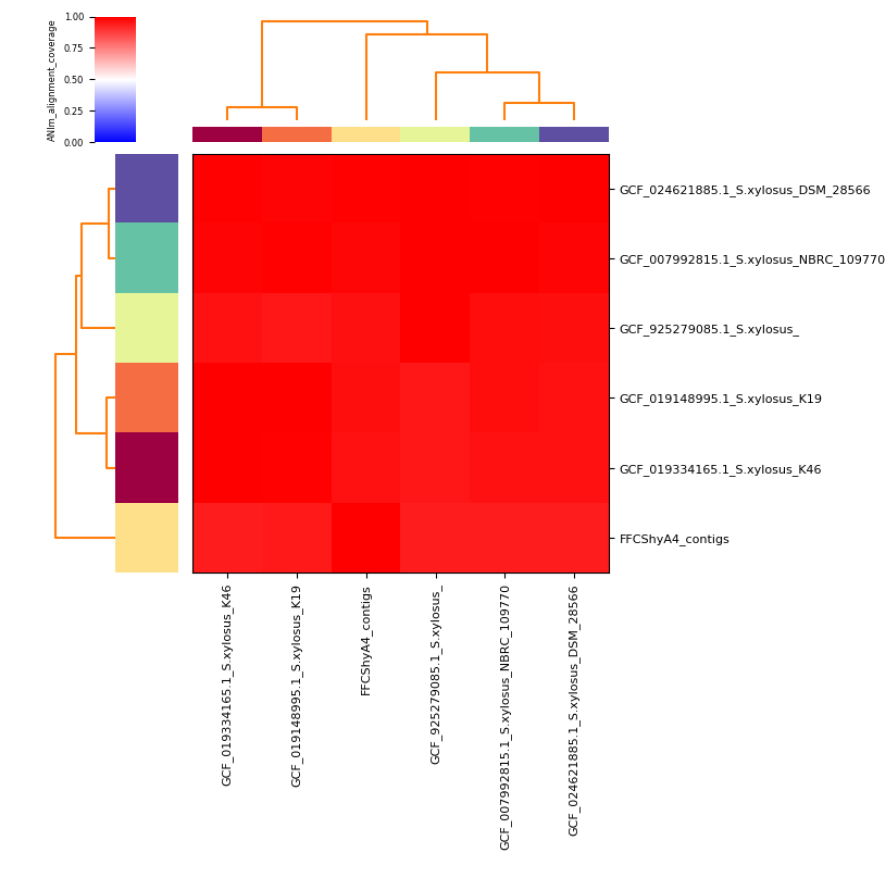


**Figure 2**. Phylogenetic tree based on TYGS results. Branch lengths are scaled in terms of GBDP (genome BLAST distance phylogeny method) distance; numbers below branches are GBDP pseudo-bootstrap support values from 100 replications. Leaf labels are annotated by affiliation to (1) species; (2) subspecies clusters; (3) genomic G+C content (min 32.48 – 33.29); (4) *δ* values (min 0.085 – 0.194); (5) overall genome sequence length (2.5 – 3.1Mb); (6) number of proteins (2469 – 2959).


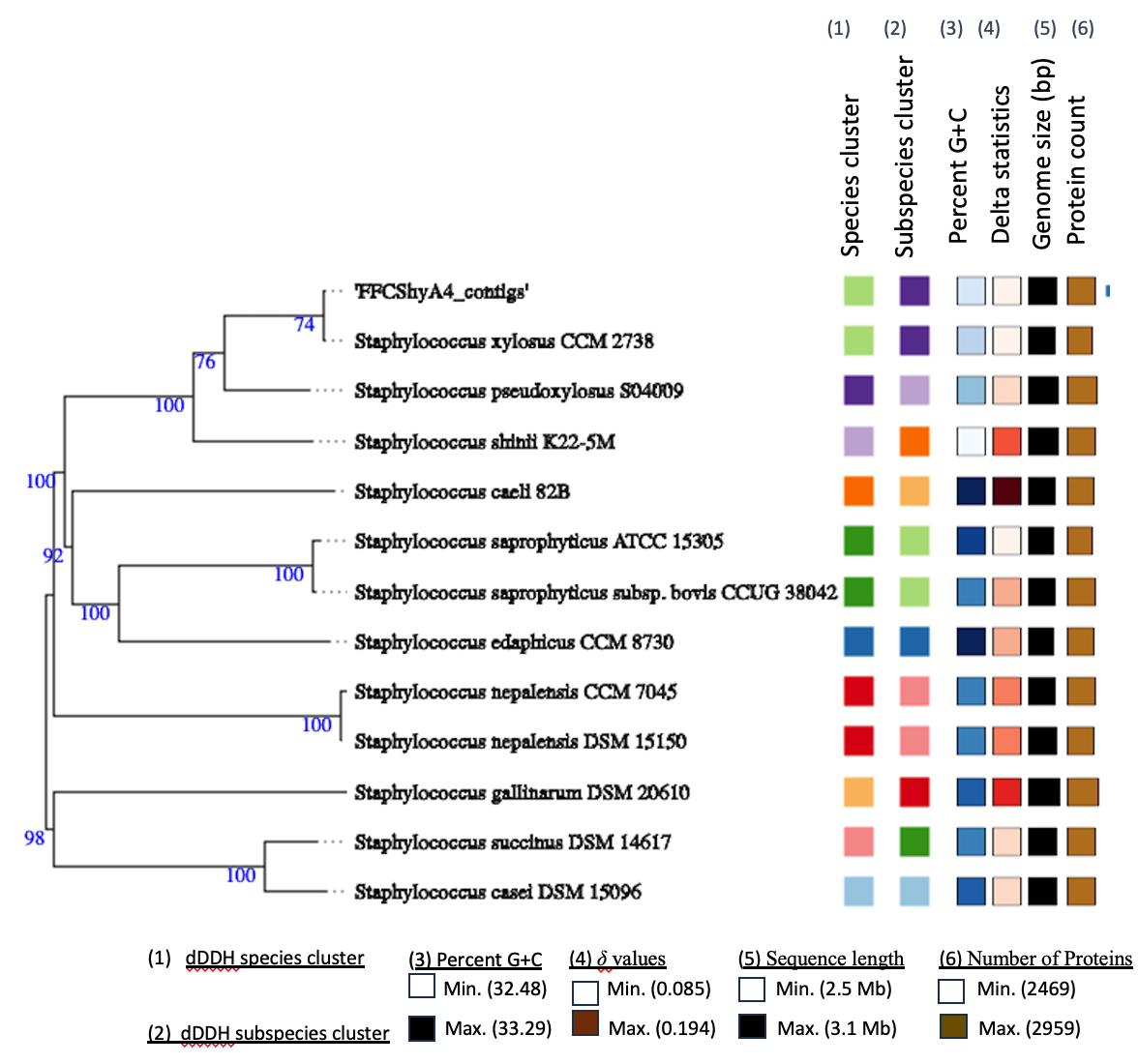


**Figure S3.** The number of genes and categories based on KEGG annotation.


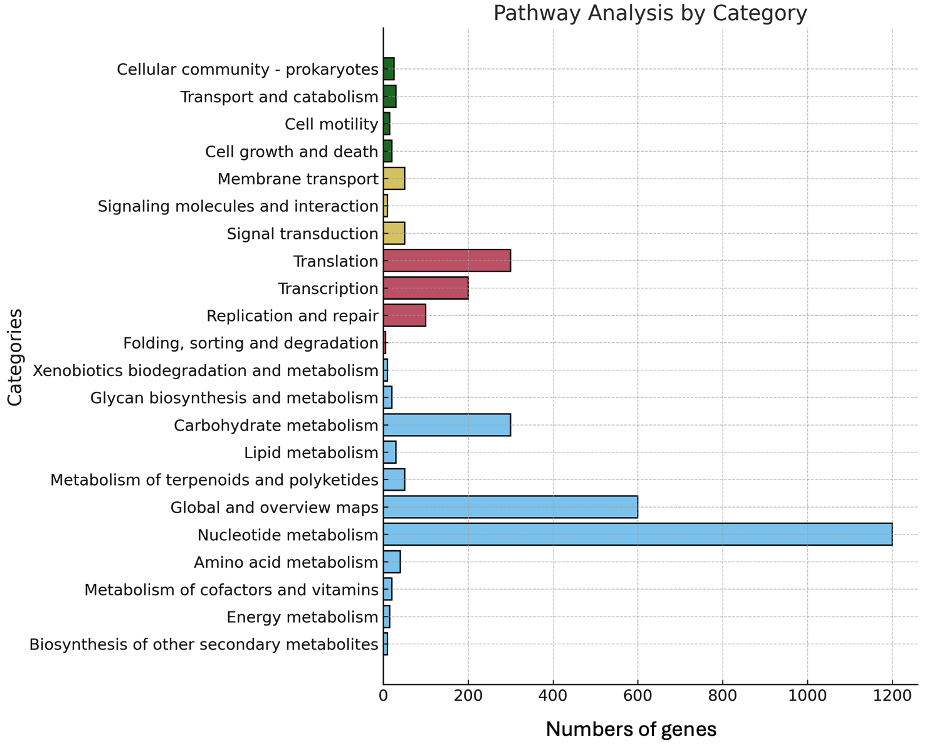


Numbers of genes

**Figure S4.** Virulence genes detected by PCR. Legend: M-molecular marker, 1- control negative, 2- S. aureus ATCC1026; 3- *S. aureus* ATCC43300; 4-*S. xylosus* FFCShyA4.

(D). *sdr*E

**Figure S5.** Blood agar plate showing hemolytic activity (zone of inhibition) of S. aureus ATCC 1026, S. aureus ATCC 43300, and S. xylosus FFCShyA4.

Zone of inhibition

**Figure S6.** Antimicrobial resistance gene families, resistance mechanisms, and drug classes of FFCShyA4 based on genome analysis. The top panel shows the distribution of AMR genes, highlighting frequent occurrences of ribosomal protection proteins and antibiotic efflux pumps. The middle panel categorizes resistance mechanisms, with antibiotic target alteration and efflux being predominant. The bottom panel represents resistance across different antibiotic classes, with high resistance noted for macrolides, tetracyclines, and fluoroquinolones.


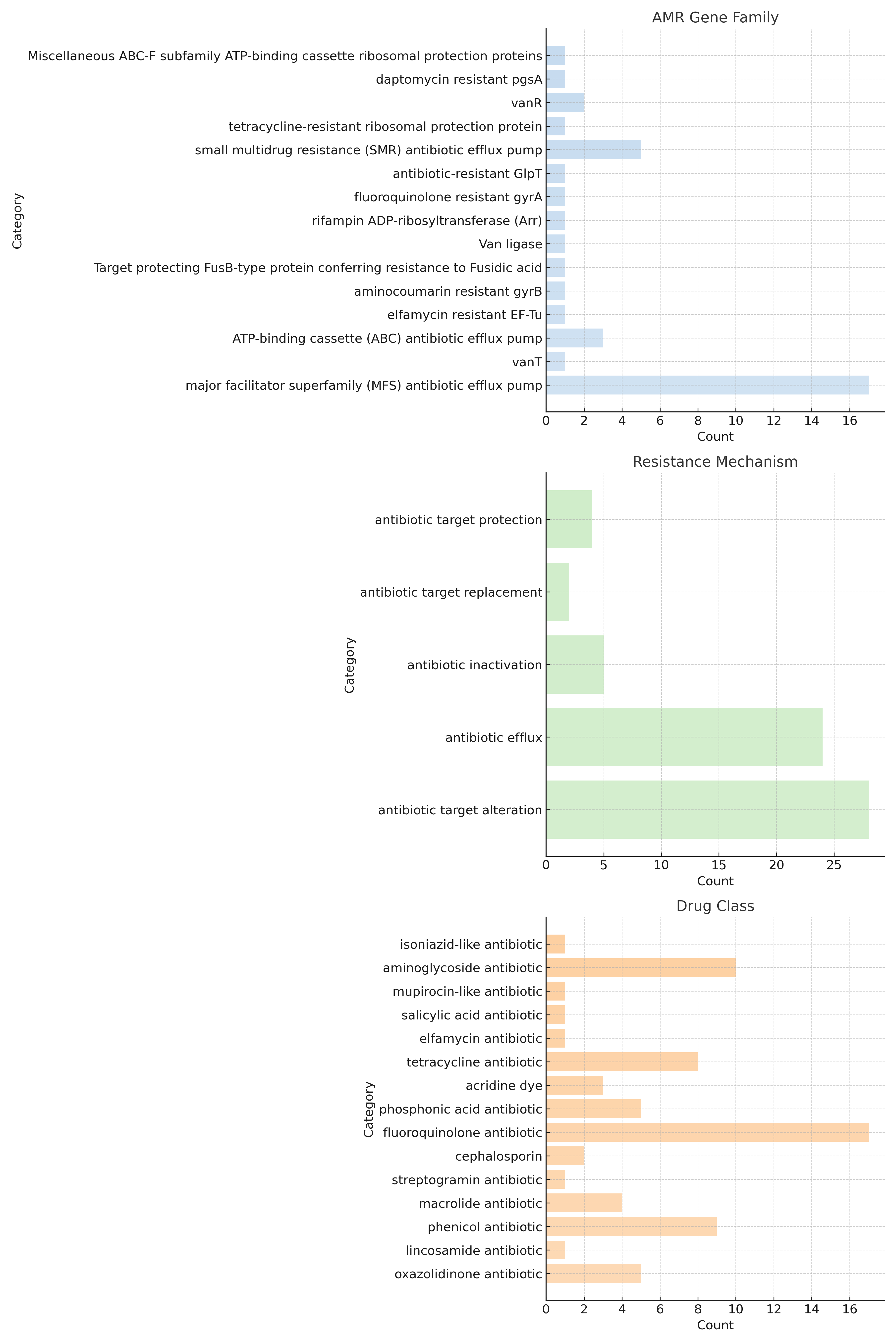


**Figure S7**. *In vitro* antibiotic resistance profile. Radar plot indicating the class and number of antibiotics for *S. xylosus* FFCShyA4 compared with *S. aureus* ATCC1026 and *S. aureus* ATCC43300.

References:

Javid, F., Taku, A., Bhat, M. A., Badroo, G. A., Mudasir, M., and Sofi, T. A. (2018). Molecular typing of *Staphylococcus aureus* based on coagulase gene. *Veterinary world*, *11*(4), 423–430. <https://doi.org/10.14202/vetworld.2018.423-430>

Kumar, R., Yadav, B. R., and Singh, R. S. (2010). Genetic determinants of antibiotic resistance in Staphylococcus aureus isolates from milk of mastitic crossbred cattle. *Current Microbiol.*, *60*(5), 379–386. <https://doi.org/10.1007/s00284-009-9553-1>.

Zhang, K., McClure, J.-A., Elsayed, S., Louie, T., and Conly, J.M. (2005). Novel Multiplex PCR Assay for Characterization and Concomitant Subtyping of Staphylococcal Cassette Chromosome *mec* Types I to V in Methicillin-Resistant *Staphylococcus aureus*. *J. Clin. Microbiol.* *43*, 5026–5033. <https://doi.org/10.1128/jcm.43.10.5026-5033.2005>.
